# The Role of Polymer Encapsulation in Optimizing Donor–Acceptor Organic Nanoparticles for Efficient Cancer Phototherapy

**DOI:** 10.3390/ijms27135863

**Published:** 2026-06-29

**Authors:** Yulia A. Isaeva, Dmitry O. Balakirev, Anastasia A. Vetyugova, Maxim E. Stepanov, Michael D. Khitrov, Nikita S. Saratovsky, Mikhail V. Zolotov, Tatyana V. Egorova, Polina A. Demina, Roman A. Akasov, Yuriy N. Luponosov

**Affiliations:** 1 Enikolopov Institute of Synthetic Polymeric Materials of the Russian Academy of Sciences, Profsoyuznaya St. 70, Moscow 117393, Russia; 2 Institute of Physics, Technology and Information Systems, Moscow Pedagogical State University, Malaya Pirogovskaya St. 29/7, Building 1, Moscow 119991, Russia; 3 Petrovsky National Research Center of Surgery, Moscow 119991, Russia; 4 Shemyakin & Ovchinnikov Institute of Bioorganic Chemistry of the Russian Academy of Sciences, Moscow 117997, Russia

**Keywords:** organic dyes, nanoparticles, photodynamic therapy, photothermal therapy, donor–acceptor molecules, encapsulation

## Abstract

Donor–acceptor (D–A) molecular systems are gaining increasing attention in cancer imaging and phototherapy due to their tunable optical properties and high photosensitizing efficiency. Encapsulation of such D–A molecules in nano-sized polymeric carriers can enhance the efficiency of antitumor therapy by passive tumor accumulation and controlled drug release. Here, we synthesized two D–A molecules—TTDCV and TTInd—based on triphenylamine with thiophene π-spacers and electron-withdrawing dicyanovinyl or indene-1,3-dione moieties. These molecules were used to preparate nanoparticles (NPs) via nanoprecipitation with amphiphilic polymers—poly(ethylene glycol)-block polylactide methyl ether (PEG-b-PLA) and polyethylene oxide-polypropylene oxide (PEO-PPO-PEO, Pluronic^®^ F-127). The resulting NPs had spherical morphology, core–shell structure and a tunable mean size (66–139 nm), depending on the polymer type used. Photothermal and photodynamic properties of the NPs were confirmed by intracellular reactive oxygen species generation and efficient heating even under 530 nm low dose irradiation (1 J/cm^2^), leading to substantial in vitro cytotoxicity against Sk-Br-3 and MCF-7 human breast cancer cells. Pluronic-encapsulated systems showed the strongest effect, reducing IC_50_ values down to 0.99 µg/mL and achieving phototoxicity indices up to 22, accompanied by increased intracellular accumulation studied by confocal microscopy and flow cytometry. This study establishes relationships between molecular design, encapsulation approaches, and the biological performance of nanoparticles, enabling the rational engineering of D–A-derived nanotherapeutics for precision cancer treatment.

## 1. Introduction

In recent decades, significant efforts have been devoted to developing advanced strategies for cancer diagnostics and therapy [1,2,3]. Among these, phototherapy has emerged as a promising and minimally invasive approach due to its simplicity, high selectivity, and therapeutic efficiency [4]. Nevertheless, the search for ideal phototherapeutic agents remains ongoing [5]. Such agents must fulfill several criteria, including selective tumor accumulation, stability under physiological conditions, high photostability, tunable absorption profiles, and efficient reactive oxygen species (ROS) generation with minimal side effects [6,7,8]. Therefore, the rational molecular design of photosensitizers (PSs) with optimized properties remains highly relevant.

Recent research has focused on the development of nanoparticles (NPs) incorporating organic PSs with enhanced optical and photochemical performance [9,10,11,12]. Among various approaches, donor–acceptor (D–A) organic semiconductors have attracted growing attention due to their broad applicability in organic electronics, including organic light-emitting diodes [13], solar cells [14,15], field-effect transistors [16], etc. Simultaneously, their favorable properties—such as biocompatibility, low toxicity, and ease of molecular tuning—have positioned them as promising candidates for biomedical applications, opening avenues for interdisciplinary research at the interface of biology and organic electronics.

One of the key advantages of D–A molecular systems is the high degree of tunability of their properties through rational molecular design. Intramolecular D–A interactions, also referred to as intramolecular charge transfer (ICT), reduce the resulting energy gap between the highest occupied molecular orbital (HOMO) and the lowest unoccupied molecular orbital (LUMO) [17,18]. Furthermore, the electronic nature of the donor and acceptor moieties, along with the degree of π-conjugation (i.e., orbital overlapping), governs the distribution of frontier orbitals and critically influences the optical and electrochemical properties of such materials [19]. Precise molecular engineering enables fine tuning of absorption and emission characteristics [20,21], enhancement of photostability [22], regulation of reactive oxygen species (ROS) generation [23,24], optimization of non-radiative relaxation pathways, and modulation of photothermal conversion efficiency [25]. These features are particularly attractive for biomedical applications, where a balance between fluorescence imaging, photodynamic activity, and photothermal performance is often required [26]. Moreover, rational molecular design allows absorption and emission bands to be shifted toward the red and near-infrared (NIR) spectral regions, which are favorable for biological applications due to reduced tissue scattering and deeper light penetration [27,28].

The rapid progress in the molecular design of D–A organic semiconductors for optoelectronic applications has laid a solid foundation for their biomedical applications. For example, fluorescent D–A-based materials have already demonstrated potential in bioimaging [29,30], while NIR-absorbing D–A systems are being actively explored for PDT and PTT and photoacoustic imaging [31,32]. However, challenges such as high hydrophobicity and limited physiological stability continue to hinder their biomedical implementation [33]. Nanotechnology offers viable solutions to these limitations. One particularly effective approach involves nanoprecipitation using amphiphilic polymers as encapsulation matrices, enabling the fabrication of stable, water-dispersible NPs while preserving the favorable photophysical properties of the encapsulated chromophores [34]. Importantly, the size and shape of NPs play critical roles in determining their biological performance, influencing circulation time, cellular uptake, and tumor accumulation via the enhanced permeability and retention (EPR) effect [35,36]. Despite the growing number of reports on D–A-based nanomaterials for biomedical use, systematic investigations into how molecular structure and encapsulation strategies jointly influence NPs properties and therapeutic outcomes remain scarce. Such studies are essential for identifying optimal combinations of molecules and carrier systems to achieve high-performance PS agents.

**Figure 1:**
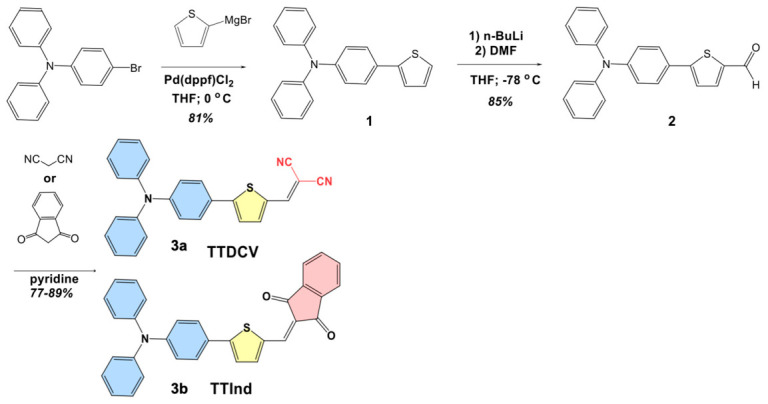
Synthetic scheme of TTDCV and TTInd molecules.

To address these knowledge gaps, we conducted a comprehensive study of two PSs with D–A molecular structure, TTDCV and TTInd (Figure 1), and their NPs form. In both cases, the electron-donor moiety is represented by triphenylamine linked via a thiophene π-conjugated spacer with either a dicyanovinyl or an indene-1,3-dione electron-withdrawing moiety. While structurally related compounds have previously been explored as PDT and PTT agents [37,38], no comparative study to date has assessed their performance across multiple delivery formats. Likewise, numerous studies have demonstrated that the therapeutic performance of organic photosensitizers can be significantly altered by the delivery format, including polymeric NPs [39], micelles [40], liposomes [41,42], and carrier-free NPs [43]. However, direct comparisons of different nanoparticle formulations based on the same D–A molecular scaffold remain limited.

Herein, we present a comparative analysis of properties and phototherapeutic efficacy for TTDCV and TTInd in three distinct forms (Figure 1): (1) aqueous dispersions of polymer-free NPs and polymer-encapsulated NPs prepared using (2) poly(ethylene glycol)-block-polylactide methyl ether (PEG-b-PLA) and (3) polyethylene oxide-polypropylene oxide (Pluronic^®^ F-127). This work provides critical insight into how molecular design and encapsulation strategy influence nanoparticle behavior and biological performance, laying the groundwork for the rational design of D–A-based nanotherapeutics for cancer treatment.

## 2. Results and Discussion

### 2.1. Synthesis of D–A Molecules

Although NIR-absorbing photosensitizers are generally preferred for in vivo phototherapy because of their deeper tissue penetration, visible-light-responsive donor–acceptor molecules remain highly valuable for mechanistic studies and treatment of superficial or accessible tumors [44,45]. D–A chromophores absorbing in the green-to-red spectral region can still exhibit efficient photothermal conversion and ROS generation under low-power irradiation. Moreover, such molecules provide important model platforms for investigating how molecular structure and nanoencapsulation affect aggregation behavior, excited-state relaxation pathways, colloidal stability, and biological activity. Therefore, TTDCV and TTInd were selected as representative D–A molecules.

The synthesis of TTDCV and TTInd was previously reported by our group [46] and involves a straightforward three-step sequence (Figure 1). First, diphenyl[4-(2-thienyl)phenyl]amine (**1**) was prepared via Kumada cross-coupling reaction between 4-bromotriphenylamine and 2-thienylmagnesium bromide in yield of 81%. In the second step, the corresponding aldehyde (**2**) was obtained through formylation of compound **1** using dry DMF and *n*-butyllithium in yield of 85%. Finally, the target D–A molecules TTDCV and TTInd were synthesized via Knoevenagel condensation of aldehyde 2 with malononitrile or 1,3-indandione, respectively, affording the desired chromophores in high yields (89% and 77%).

### 2.2. Preparation and Characterization of NPs

#### 2.2.1. Preparation of NPs and Their Characterization by Size and Shape

**Figure 2:**
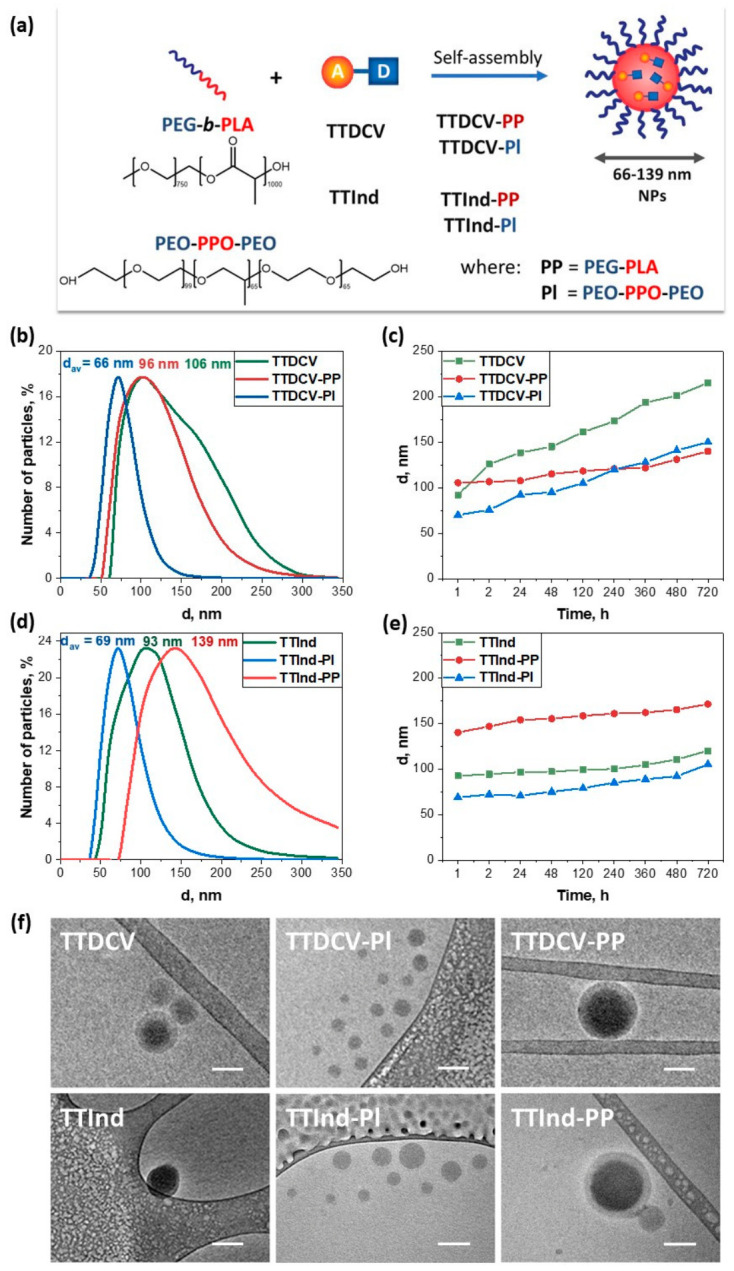
(**a**) Illustration of TTDCV and TTInd NPs dispersed in deionized water, preparation through self-assembly method; (**b**,**d**) size distribution of TTDCV (**b**) and TTInd (**d**) NPs obtained by DLS; (**c**,**e**) kinetics of TTDCV (**c**) and TTInd (**e**) NP size growth; (**f**) morphology of prepared TTDCV and TTInd NPs obtained by cryoTEM with a scale bar of 100 nm.

NPs based on TTDCV and TTInd PSs were prepared via two distinct approaches: (1) nanoprecipitation from a dimethyl sulfoxide (DMSO) solution into water, yielding the polymer-free NPs dispersion and (2) encapsulation within two amphiphilic polymer matrices—poly(ethylene glycol)-block-poly(lactic acid) methyl ether (PEG-b-PLA; PP) or poly(ethylene oxide)-poly(propylene oxide)-poly(ethylene oxide) (Pluronic^®^ F-127; Pl) (Figure 2a). These polymers were selected for their high biocompatibility and proven ability to stabilize hydrophobic NPs in aqueous environments. In particular, PEG-based block copolymers are widely known to reduce protein adsorption and prolong blood circulation times, making them widely used in biomedical nanomaterials design [47]. Moreover, the amphiphilic character of PP and Pl polymers may facilitate efficient encapsulation of hydrophobic small molecules, improving their colloidal stability and bioavailability.

Dynamic light scattering (DLS) measurements revealed that Pl-encapsulation afforded the smallest and most monodisperse NPs (d_av_ ≈ 66 nm for TTDCV-PI and ≈ 69 nm for TTInd-PI) (Figure 2b,d). In contrast, polymer-free NPs exhibited the broadest size distributions and the largest mean diameters, while usage of PP showed molecule-dependent effects: for TTDCV size decreased modestly (~10 nm) upon encapsulation, whereas for TTInd the d_av_ values increased from ≈93 nm up to ≈139 nm. In general, diameters of around 50–200 nm are considered the most promising for antitumor therapy as they can accumulate in tumors due to the enhanced permeability and retention effect [48] and enter cells via endocytosis [49]. This also contributes to their colloidal stability, which is critical for practical implementation. Zeta potential (ζ) measurements revealed negative surface charges for all NPs formulations. Polymer-free TTDCV and TTInd NPs exhibited ζ-potentials of −16.5 and −19.3 mV, respectively, indicating a slightly higher surface charge density for TTInd NPs. Encapsulation within PEG-b-PLA and Pluronic^®^ F-127 resulted in a decrease in the absolute ζ-potential values, particularly for Pluronic^®^ F-127 formulations, whose ζ-potentials approached neutrality. This effect can be attributed to partial shielding of the NP surface charge by the hydrated PEG/PEO-rich polymer shell. Consequently, the colloidal stability of the polymer-encapsulated NPs is governed predominantly by steric stabilization rather than electrostatic repulsion, in agreement with previous reports for PEGylated and Pluronic-based nanocarriers [50,51].

The stability of NPs over time strongly depends on the D–A molecule used. It was found that the particle size increases significantly over time in the case of TTDCV, and only encapsulation with PP significantly slows down the aggregation of NPs. Whereas in the case of TTInd, almost all forms of NPs have a fairly high stability (Figure 2c,e). A similar trend in colloidal stability over time was confirmed for TTDCV and TTInd NPs in saline/fetal bovine serum (FBS) mixtures containing 1% and 10% FBS (Figure S4). Thus, the choice of the appropriate polymer matrix may improve the stability of NPs, which is important for efficient therapeutic agents.

Cryogenic transmission electron microscopy (Cryo-TEM) images demonstrate predominantly spherical morphology for all samples (Figure 2f). It is noteworthy that some NPs exhibit internal contrast that corresponds to either a “core–shell” structure or a structure with a gradient density suggesting either phase separation or changes in packing density. The Cryo-TEM results confirmed the DLS measurement data, indicating that the mean size of the NPs is around 100 nm, which is attractive for biomedical applications.

#### 2.2.2. Optical and Photophysical Properties of NPs

**Figure 3:**
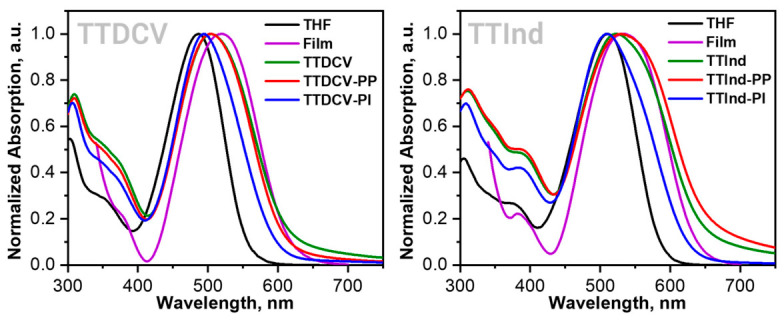
Normalized absorption spectra of TTDCV and TTInd in THF solution, film, polymer free NPs (NPs), PP-encapsulated (PP) and Pl-encapsulated (Pl) NPs.

Optical properties of the PSs were examined for dilute THF solutions (10^−5^ M), polycrystalline films and aqueous dispersions of polymer-free and polymer-encapsulated NPs (PP and Pl) (Figure 3, Figures S5 and S6 and Table 1).

In THF, TTDCV and TTInd show a pronounced blue-green-region absorption spectra with maxima (λ_abs_) at 487 nm, and 511 nm, respectively (Figure 3, Table 1), along with the corresponding red-shifted photoluminescence peaks at 657 nm and 669 nm, respectively (Figure S5). Going from dilute THF solutions to polycrystalline films revealed bathochromic shifts of the λ_abs_ (up to 520 nm for TTDCV and to 532 nm for TTInd) and the spectra broadening, reflecting stronger π–π interactions and increased energetic disorder in the condensed phase caused by aggregation processes.

NP formation induces distinct spectral changes that depend on both the molecular structure and the encapsulation matrix. In general, the optical properties of the NPs were directly referred to the average diameter distribution of NPs, dedicated to the nanoprecipitation conditions. Pl-encapsulated NPs (smallest d_av_) yielded absorption spectra most similar to the molecular THF solutions (Figure 3, Table 1). In contrast, the PP-encapsulation resulted in optical properties close to the polymer-free NPs (Table 1). A similar spectral distribution was observed for the polymer-free and PP-encapsulated TTDCV NPs, while both the spectra had hypsochromic shifts compared to the film. In the case of TTInd NPs, both polymer-free and PP-encapsulated NPs were characterized by the slight lower λ_abs_ values together with pronounced bathochromic shifts of the λ_onset_ compared to the film. This in a good agreement with the fact that TTInd-PP NPs were found to be in a much more aggregated form compared to pure TTInd NPs.

The photostability of the NPs was assessed under continuous irradiation with green LED light for 30 min (Figure S7). The absence of changes in the shape and intensity of the absorption spectra indicates equally high photostability for both polymer-free NPs and NPs encapsulated in an amphiphilic polymer matrix.

The release behavior of TTDCV and TTInd from polymeric NPs was systematically investigated under physiological conditions. The release profiles were monitored over 72 h by recording the characteristic absorption spectra of the dyes at predetermined time points (Figure S8). Changes in spectral intensity were used to evaluate the extent of dye release from the nanoparticle core into the medium. The results demonstrate that the release kinetics of TTDCV and TTInd are determined by both the nature of the encapsulated molecule and the type of amphiphilic polymer matrix. Overall, Pluronic-based NPs are characterized by more stable dye retention compared to PEG-PLA, while TTInd exhibits a more pronounced release tendency compared to TTDCV (Figure S8).

#### 2.2.3. DFT Calculations

**Figure 4:**
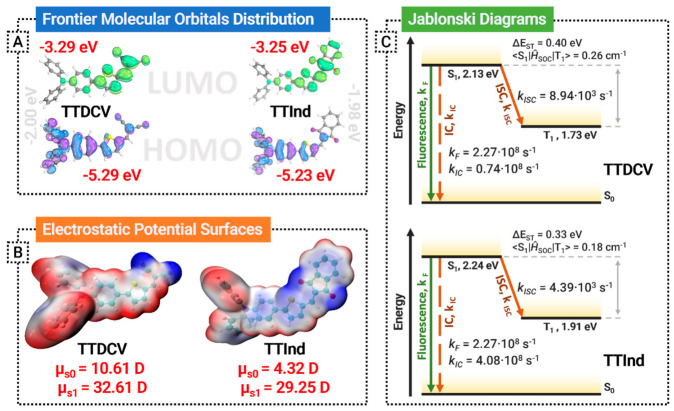
(**A**) DFT-calculated frontier orbital distribution and energies; (**B**) electrostatic potential surfaces and dipole moments; (**C**) Jablonski diagrams of TTDCV and TTInd.

To gain a deeper understanding of the molecular properties of the compounds under study, we performed density functional theory (DFT) calculations, the results of which can be roughly compared with the properties of the corresponding nanoparticles. After performing a conformational search and selecting one structure for each compound, all subsequent calculations were performed at the PBE0-D4/def2-TZVPPD//PBE0-D4/def2-SVP theory level (see Supplementary Materials and Table S1 for details). Using the continuum solvation model CPCM(Acetonitrile), good agreement of the frontier orbital energies with experimental data was achieved [46], confirming the adequacy of the model choice (Figure 4A). Using the mentioned level of theory, dipole moment values and electrostatic potential (ESP) surface were also obtained (Figure 4B). ESP indicates significant molecular polarization characteristic of D-A chromophores, with a more diffuse electron density on the triphenylamine-thiophene moiety (red region) and a more dense electron density on the acceptor substituent (blue region).

We then performed Excited State Dynamic calculation [52] (see Supplementary Materials for details), which allowed us to obtain model rate constants for fluorescence S_1_ → S_0_ k_F_, non-radiative relaxation (internal conversion, IC) S_1_ → S_0_ k_IC_, and intersystem crossing (ISC) S_1_ → T_1_ k_ISC_ (Figure 4C). The fluorescence and non-radiative relaxation constants are in good agreement with experimental data [46] (taking into account the lack of an environment model) and give adequate values of the luminescence quantum yields Φ_PL_ 76% and 36% for TTDCV and TTInd, respectively. With the IC rate constant for TTInd is more than five times greater than that for TTDCV (4.08 × 10^8^ vs. 0.74 × 10^8^ s^−1^, respectively), we should expect more efficient photothermic energy conversion in the case of TTInd.

Moving to the triplet-state dynamics, we have calculated singlet-triplet gap energies ∆E_ST_ using so-called ∆SCF approach, which usually shows accuracy comparable to high-level methods [53,54]. The predicted values of ∆E_ST_ 0.40 and 0.33 eV gave us the triplet level energies of 1.73 and 1.91 eV for TTDCV and TTInd, respectively. The energies obtained for the triplet levels of both compounds are well suited for efficient energy transfer to oxygen, exceeding both the energy of its first (0.98 eV) and the higher-lying excited states (1.65 eV) [55]. It should be noticed that in case of TTDCV the triplet-level energy matches better with the second excitation energy of oxygen. As was stated above, k_ISC_ were also predicted as being 8.94 × 10^3^ and 4.39 × 10^3^ s^−1^ for TTDCV and TTInd, respectively. Since for TTDCV the value is more than twice higher than that of TTInd, a higher population of the triplet state can be expected, which, together with its better energy match for oxygen excitation, may result in more efficient ROS generation in case of TTDCV.

#### 2.2.4. Photothermal Properties of TTDCV and TTInd NPs

**Figure 5:**
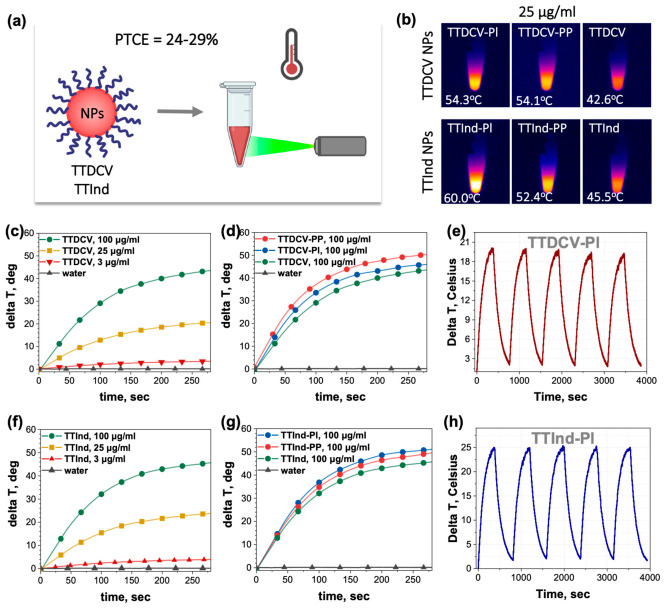
Photothermal conversion efficiency determination. (**a**) General scheme of photothermal property evaluation of TTDCV and TTInd NPs; (**b**) infrared thermal images of 25 μg/mL TTDCV and TTInd NPs and water under 530 nm LED (0.5 mW/cm^2^); (**c**,**f**) photothermal response of NPs with different concentrations of TTDCV (**c**) and TTInd (**f**); (**d**,**g**) different forms of TTDCV (**d**) and TTInd (**g**) NPs (100 μg/mL) under 530 nm laser irradiation (0.5 W/cm^2^) for 6 min; (**e**,**h**) temperature elevation curves of 5 heating–cooling cycles of 20 μg/mL TTDCV (**e**) and TTInd (**h**) NPs under 530 nm LED (0.3 W/cm^2^).

Suppressing the fluorescence of NPs promotes the internal conversion process of the excited state, whereby absorbed light energy is converted into heat. As a result of this process, heat is released, making NPs effective for photothermal therapy, as they can heat surrounding tissues when irradiated. Therefore, TTDCV and TTInd NPs can presumably act as PTT agents, generating heat in aqueous media when irradiated with light. To confirm the photothermal properties of the NPs and investigate photothermal conversion, the dispersions were irradiated with a 530 nm laser (0.5 W/cm^2^), and temperature changes were tracked using an infrared thermal imaging camera (Figure 5). Figure 5c and Figure 5d show the temperature distributions in TTDCV and TTInd NP dispersions, respectively, of different concentrations after 360 s of irradiation. The control without NPs (pure deionized water) shows no observable temperature changes. Even at a relatively low concentration of 25 μg/mL, the samples reach a lethal temperature of >41 °C within 300 s, and at a higher concentration of 100 μg/mL, the temperature can exceed 70 °C (Figure 5d). The photothermal conversion efficiency (PTCE) (*η*) of the TTDCV and TTInd NPs was calculated at a concentration of 25 μg/mL and is presented in Table S2 (Supplementary Materials). In general, the obtained values of *η* were similar for all forms (Table S2, Supplementary Materials). The ability of NPs to convert light into heat may be due to the molecular structure of the components and the NP aggregation characteristics, where slightly higher *η* values for TTInd may be associated with higher values of the IC rate constant as compared to TTDCV. As Figure 5e,h show, the NPs retained their excellent photothermal conversion ability even after five laser illumination cycles.

It should be noted that thermal imaging camera evaluates integral temperature changes throughout the suspension volume. At the intracellular level, however, the NPs individually heat to a higher local temperature, which then dissipates through the tissue [56]. Therefore, the local intracellular heating is higher than the measured integral temperature. Moreover, the ideal option would be to eliminate the tumor cells that took up the NPs with the photoinduced heating, while keeping the integral temperature below 40 °C to avoid burns.

#### 2.2.5. In Vitro ROS Generation and Cytotoxicity of NPs

**Figure 6:**
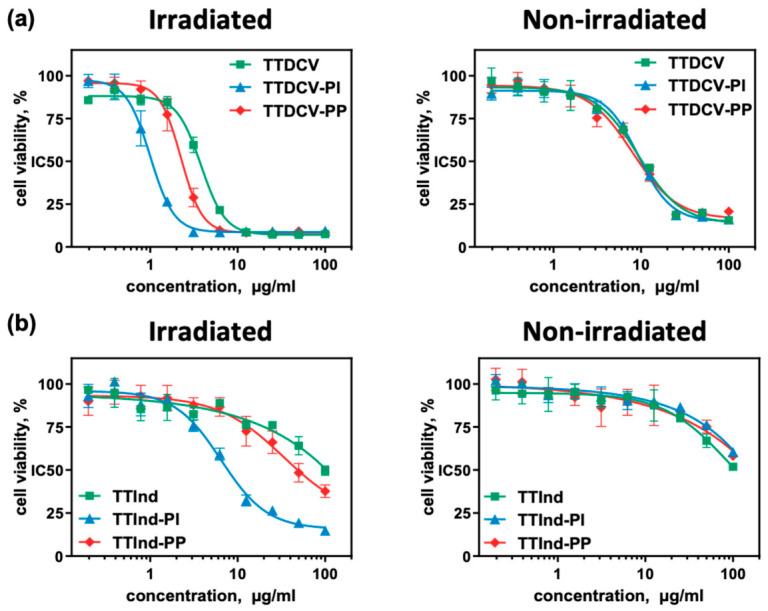
(**a**,**b**) Cell viability of human breast cancer MCF-7 cells depending on the concentration of TTDCV (**a**) and TTInd (**b**) NPs (μg/mL) under irradiated (530 nm, 1 J/cm^2^) and non-radiated conditions. The data are the mean ± SD. IC_50_—half-max inhibitory concentration; PI—phototoxicity index, PI = IC_50 irradiated_/IC_50 non-irradiated_.

The antiproliferative activity of TTDCV and TTInd NPs was investigated using human breast adenocarcinoma Sk-Br-3 and MCF-7 cells and lung fibroblast WI-26 and human melanoma Mel Z cells under light and dark conditions (Figure 6 and Figure S9, Table S3). It was shown that TTDCV NPs were more toxic to cells than TTInd NPs after a 72 h incubation in the dark. Light exposure (530 nm, 1 J/cm^2^) resulted in the increase of cytotoxicity for TTDCV NPs with the phototoxicity indexes (PI) of 2.4–5.4 but not for TTInd NPs with PI ~1. However, in both cases, polymer-encapsulated NPs exhibited significantly enhanced cytotoxicity with a phototoxicity index ranging from 2 to 22 (Table 2). Thus, the introduction of polymers reduced the IC_50_ in Sk-Br-3 cells by 3.6 and 4.3 and in MCF-7 cells by 3.8 and 1.7 times for TTDCV-Pl and TTDCV-PP, respectively, compared to polymer-free TTDCV. It should be noted that the polymer-free TTInd NPs were initially of low toxicity in comparison to TTDCV NPs; however, the encapsulation in TTInd-Pl, but not in TTInd-PP, resulted in the sharp increase of cytotoxicity (>20 folds) that demonstrated the potential of PI for cancer cell sensibilization in therapy.

**Figure 7:**
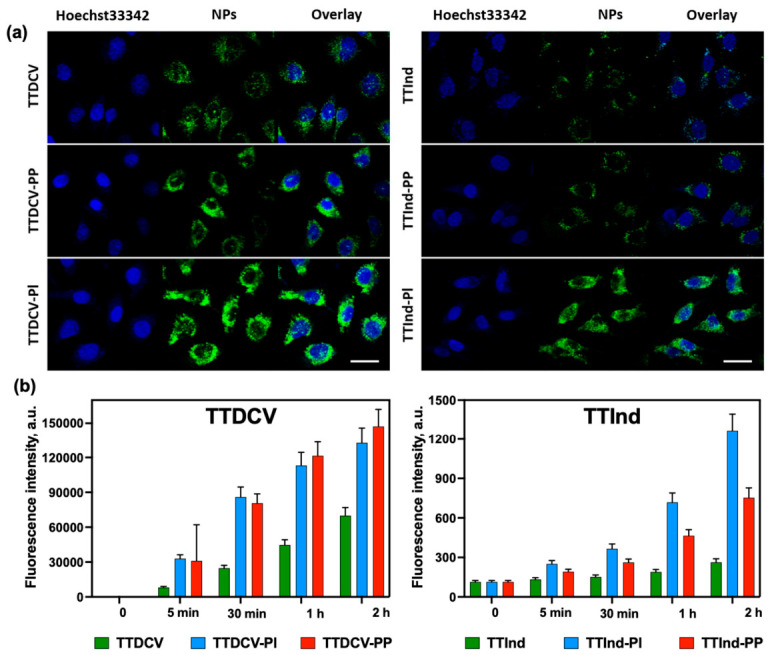
(**a**) Intracellular accumulation of TTDCV and TTInd NPs in human breast cancer Sk-Br-3 cells at 10 µg/mL (confocal microscopy data at a scale bar of 20 µm). Cell nuclei were additionally stained with Hoechst 33342. Fluorescence was analyzed at 405 nm (Hoechst 33342, in blue) and 488 nm (dyes, in green). (**b**) The accumulation of TTDCV and TTInd in human breast carcinoma MCF-7 cells is also shown at 10 µg/mL (flow cytometry data).

The intracellular distribution and total accumulation of the NPs were studied using confocal microscopy and flow cytometry techniques (Figures S7 and S10, Supplementary Materials). It was found that all TTDCV-based NPs are better accumulated in cells in comparison to TTInd (Figure 7a). These findings correlated to cytotoxicity data, which demonstrated the superior toxicity of TTDCV. It should be noted that both polymers enhanced the intracellular accumulation, and encapsulation with Pluronic facilitated superior accumulation of both TTDCV and TTInd. This could be explained with the smaller size of the NPs (66–69 nm), which is preferable for endocytosis. The dot-like intracellular fluorescence confirmed the endocytosis of the NPs, and the uptake of the NPs into lysosomes could be proposed. The accumulation of the comparatively large TTInd-PP NPs (139 nm) is practically no different from the accumulation of the unencapsulated form, which explains the reduced cytotoxicity of TTInd-PP NPs compared to TTInd-Pl.

**Figure 8:**
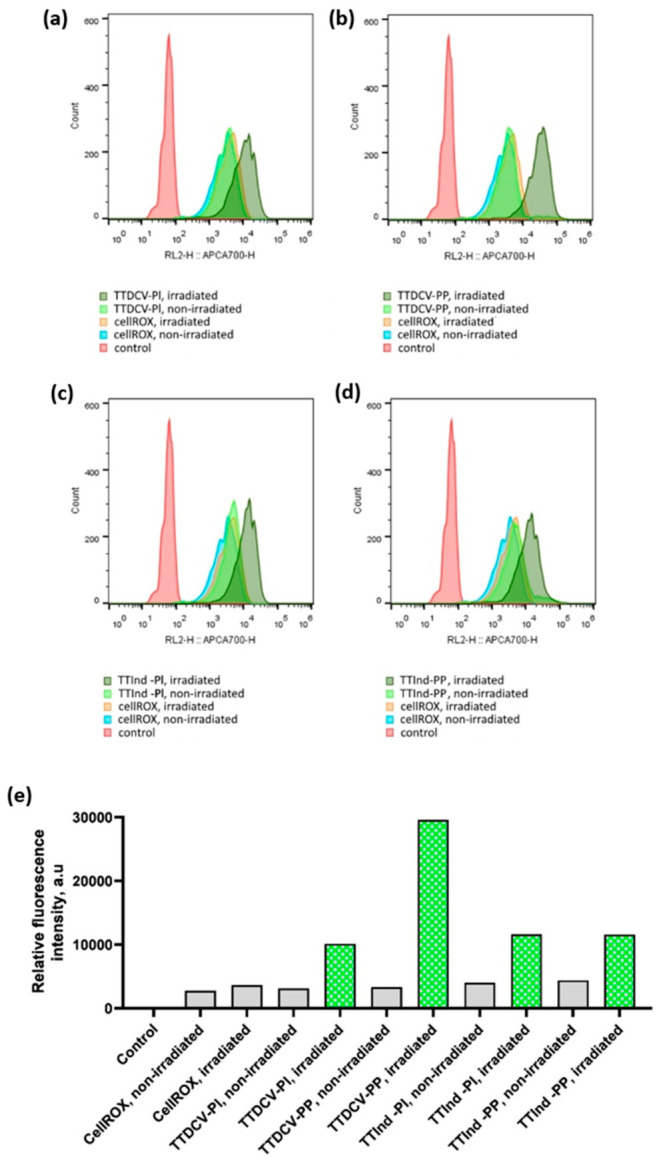
(**a**) CellROX Deep Red staining of human breast adenocarcinoma MCF-7 cells: TTDCV-PI; (**b**) TTDCV-PP; (**c**) TTInd-PI; and (**d**) TTInd-PP. Median values of CellROX Deep Red fluorescence: (**e**) flow cytometry data, with 10,000 counts per each sample. Green color indicates the irradiated samples.

The intracellular production of ROS following irradiation was quantified using a deep red CellROX^®^ assay (Figures S11 and S12, Supplementary Materials) and flow cytometry to obtain a more reliable quantitative assessment compared to confocal microscopy. In addition, H_2_O_2_ and ascorbic acid were used as positive and negative controls, respectively (Figure S13). Using the CellROX Deep Red assay, we found clear light-induced ROS generation in all studied NPs (Figure 8). ROS generation correlated with intracellular accumulation levels; therefore, TTDCV-PP demonstrated the highest ROS generation. Interestingly, TTInd NPs generated similar ROS values to those of TTDCV-PI despite lower accumulation. Adding ascorbic acid, which has antioxidant properties, decreased ROS generation (Figure S13). Adding H_2_O_2_, however, increased ROS generation. It should be noted that ROS generation was significantly higher in TTDCV-treated cells than in TTInd-treated cells. Furthermore, the ROS generation in TTDCV-treated cells was clearly concentration-dependent (increasing the concentration from 1 µg/mL to 10 µg/mL resulted in a ~4-fold increase in ROS), whereas the ROS generation in TTInd-treated cells did not demonstrate a clear correlation (Figure S14). Therefore, the higher ROS sensitivity expected in DFT calculations was confirmed by in vitro ROS measurements. In conclusion, there was clear ROS generation in cells under 530 nm irradiation, which proves the photodynamic cell death mechanism.

Therefore, two independent pathways of light-induced toxicity of novel D–A molecules can be proposed, namely photodynamic and photothermal toxicity. Photodynamic toxicity involves the generation of ROS, while photothermal toxicity uses the heating of cells to above 42 °C, resulting in the disruption of biomolecules. Intracellular accumulation is necessary for both pathways, so encapsulating the D–A molecules in biopolymers (PEG-b-PLA or Pluronic F-127) enhances cytotoxicity.

Previous studies on D–A photosensitizers have primarily focused on maximizing ROS generation [57,58], photothermal conversion efficiency [59], or red-shifted absorption through molecular engineering of the chromophore [24,60]. In contrast, comparatively less attention has been paid to the role of NPs formulation in determining the final biological response. Our results demonstrate that phototherapeutic efficacy cannot be explained solely by the intrinsic electronic structure of the D–A molecule. Although TTDCV and TTInd possess similar D–A architectures and exhibit comparable photophysical characteristics, substantial differences in cellular uptake, ROS production, and phototoxicity were observed depending on the NPs formulation. Similar behavior has been reported for triphenylamine-based D–A photosensitizers [61,62]. Various molecular design strategies have been employed for triphenylamine-based D–A molecules to improve cellular targeting [63], enhance photothermal [64,65] and photodynamic effects [66], shift the absorption spectrum to the red and NIR ranges [31], and synergistic PDT/PTT therapy [67,68]. Furthermore, while studies frequently demonstrate the dependence of biological properties on the molecular structure of the D–A molecules, they do not evaluate how different NP formulations influence the biological response of the same molecule. The present results demonstrate that changing the encapsulation matrix alone can substantially alter cellular uptake and phototherapeutic efficacy. Nevertheless, the study is limited by its focus on in vitro experiments; further investigation requires a detailed study of biosafety, biodistribution, pharmacokinetics, etc. Furthermore, the study of interactions between D–A molecules and cells was limited to conventional toxicity and accumulation/intracellular distribution studies. However, establishing pathways at a molecular level could help to estimate the potential of D–A molecules for photodynamic therapy.

## 3. Materials and Methods

### 3.1. Materials

4-Bromotriphenylamine, thiophene, magnesium turnings, [1,1′-*bis*(diphenylphosphino)ferrocene]dichloropalladium(II) (Pd(dppf)Cl_2_), *n*-butyllithium (1.6 M solution in hexane; *n*-BuLi), malononitrile and 1,3-indandione were obtained from Merck Co. (Rahway, NJ, USA) and used without further purification. THF, DMF and pyridine were purified and/or dried according to the known techniques. In the case of column chromatography, Silica Gel 60 (Merck Co., USA) was taken. Knövenagel condensation reactions were carried out in the microwave “Discovery” reactor (CEM Co., Matthews, NC, USA), using a standard method with the open vessel option, 30 W.

### 3.2. Synthetic Procedures

Diphenyl[4-(2-thienyl)phenyl]amine (**1**) was obtained as reported elsewhere [69]. The crude product was purified by column chromatography (silica gel; eluent:hexane/toluene = 12/1) to give the pure compound **1** (12.24 g, 83%) as a white solid. M.p. = 133 °C. The ^1^H NMR values were as follows (250 MHz, CDCl_3_, δ, ppm): 7.01–7.16 (overlapping peaks, 9H); 7.20–7.32 (overlapping peaks, 6H); and 7.47 (d, 2H, J = 8.66 Hz). The ^13^C NMR values were as follows (75 MHz, CDCl_3_, δ, ppm): 122.21; 123.01; 123.75; 124.00; 124.41; 126.70; 127.97; 128.51; 129.29; 144.24; 147.17; and 147.47. The Calcd. (%) for C_22_H_17_NS were as follows: C, 80.70; H, 5.23; N, 4.28; and S, 9.79. Furthermore, the found C was 81.04 with H, 5.32; N, 4.10; and S, 9.82. The MALDI-TOF MS results were as follows: found *m*/*z* 327.1 and calcd. for [M]^+^ 327.1.

5-[4-(diphenylamino)phenyl]thiophene-2-carbaldehyde (**2**) was obtained as reported elsewhere [70]. The crude product was purified by column chromatography (silica gel; eluent: dichloromethane) to give the pure product **2** (5.10 g, 87%) as a yellow solid. M.p. = 117 °C. The ^1^H NMR (250 MHz, CDCl_3_, δ, ppm) values were as follows: 7.02–7.18 (overlapping peaks, 8H); 7.25–7.34 (overlapping peaks, 5H); 7.51 (d, 2H, J = 8.55 Hz); 7.70 (d, 1H, J = 3.96 Hz); and 9.85 (s, 1H). The ^13^C NMR values were as follows (75 MHz, CDCl_3_, δ, ppm): 122.32; 122.83; 123.84; 125.14; 126.08; 127.21; 129.46; 137.74; 141.26; 146.93; 149.10; 154.55; and 182.60. The Calcd. (%) for C_23_H_17_NOS were as follows: C, 77.72; H, 4.82; N, 3.94; and S, 9.02. Furthermore, the found C was 78.18 with H, 4.85; N, 3.90; and S, 8.88. The MALDI-TOF MS results were as follows: found *m*/*z* 355.1 and calcd. for [M]^+^ 355.1.

Below explains how {4-[5-(2,2-dicyanovinyl)-2-thienyl]phenyl}diphenylamine (TTDCV) was produced. Compound **2** (0.90 g, 2.5 mmol), malononitrile (0.25 g, 3.8 mmol) and dry pyridine (23 mL) were placed in a reaction vessel and stirred under an argon atmosphere for 1 h at a microwave-assisted reflux. After the reaction completion, pyridine was evaporated under vacuum, and the residue was dried at 1 Torr. The crude product was purified by column chromatography (silica gel; eluent: dichloromethane). Further purification included the product precipitation from its THF solution with hexane to give the pure product TTDCV (0.92 g, 90%) as a black solid. M.p. = 114 °C. The ^1^H NMR (Figure S1) (250 MHz, CDCl_3_, δ, ppm) values were as follows: 7.00–7.17 (overlapping peaks, 8H); 7.26–7.35 (overlapping peaks, 5H); 7.52 (d, 2H, J = 8.85 Hz); 7.66 (d, 1H, J = 4.27 Hz); and 7.73 (s, 1H). The ^13^C NMR (Figure S2) (75 MHz, CDCl_3_, δ, ppm) values were as follows: 113.66; 114.55; 121.67; 123.25; 124.31; 124.80; 125.48; 127.54; 129.56; 133.05; 140.38; 146.59; 149.89; 150.26; and 157.11. Calcd. (%) for C_26_H_17_N_3_S were as follows: C, 77.39; H, 4.25; N, 10.41; and S, 7.95. Furthermore, the found C was 77.55 with H, 4.32; N, 10.33; and S, 7.93. The MALDI-TOF MS results were as follows: found *m*/*z* 403.1 and calcd. for [M]^+^ 403.1.

Below explains how ({5-[4-(diphenylamino)phenyl]-2-thienyl}methylene)-1*H*-indene-1,3(2*H*)-dione (TTInd) was produced. Compound **2** (0.90 g, 2.5 mmol), 1,3-indandione (0.46 g, 3.2 mmol) and dry pyridine (27 mL) were placed in a reaction vessel and stirred under an argon atmosphere for 5 h at a microwave-assisted reflux. After the reaction completion, pyridine was evaporated under vacuum, and the residue was dried at 1 Torr. The crude product was purified by column chromatography (silica gel; eluent: dichloromethane). Further purification included the product precipitation from its THF solution with hexane to give the pure product TTInd (1.04 g, 85%) as a red solid. M.p. = 194 °C. The ^1^H NMR (Figure S3) (250 MHz, CDCl_3_, δ, ppm) values were as follows: 7.02–7.18 (overlapping peaks, 8H); 7.26–7.38 (overlapping peaks, 5H); 7.62 (d, 2H, J = 8.85 Hz); 7.73–7.78 (overlapping peaks, 2H); and 7.91–7.99 (overlapping peaks, 4H). Calcd. (%) for C_32_H_21_NO_2_S were as follows: C, 79.48; H, 4.38; N, 2.90; and S, 6.63. Furthermore, the found C was 79.68 with H, 4.43; N, 2.89; and S, 6.59. The MALDI-TOF MS results were as follows: found *m*/*z* 483.0 and calcd. for [M]^+^ 483.1.

### 3.3. General Methods

^1^H NMR spectra were recorded using a “Bruker WP-250 SY” spectrometer (Bruker Corporation, Karlsruhe, Germany), working at a frequency of 250 MHz and using a CDCl_3_ (7.25 ppm) signal as the internal standard. ^13^C NMR spectra were recorded using a “Bruker Avance II 300” spectrometer (Bruker Corporation, Germany), working at a frequency of 75 MHz. In the case of ^1^H NMR spectroscopy, the compounds to be analyzed were taken in the form of 1% solutions in CDCl_3_. In the case of ^13^C NMR spectroscopy, the compounds to be analyzed were taken in the form of 5% solutions in CDCl_3_. The spectra were then processed on the computer using the “ACD Labs” software (version 2016free). 

Elemental analysis of C, N and H elements was carried out using CHN automatic analyzer “CE 1106” (Carlo Erba, Milan, Italy). The settling titration using BaCl_2_ was applied to analyze the S element.

MALDI-TOF were registered on a “Autoflex II Bruker” (Bruker Daltonik GmbH, Germany) (resolution FWHM 18,000), equipped with a nitrogen laser (work wavelength 337 nm) and time-of-flight mass-detector working in the reflections mode. The accelerating voltage was 20 kV. Samples were applied to a polished stainless-steel substrate. Spectrum was recorded in the positive ion mode. The resulting spectrum was the sum of 300 spectra obtained at different points of the sample. 2,5-Dihydroxybenzoic acid (DHB) (Acros Organics (99%), Geel, Belgium) and α-cyano-4-hydroxycinnamic acid (HCCA) (Acros Organics (99%), Belgium) were used as matrices.

The absorption spectra of the corresponding compounds were recorded with a SILab u-Violet R (China) spectrophotometer (Beijing Beifen-Ruili Analytical Instrument (Group) Co., Ltd., Beijing, China) in the standard 10 mm photometric quartz cuvette. The absorption spectra were recorded using THF and DMSO solutions with the concentrations of 10^−5^ M. The absorption spectra of the nanoparticle dispersions of the corresponding compounds were recorded using distilled water as reference sample. All measurements were carried out at room temperature. The release kinetics of TTDCV and TTInd NPs were recorded at 0, 1, 2, 24, and 72 h at 37 °C. Progressive changes in absorbance intensity indicate dye release from the NPs matrices.

A scanning spectrofluorometer Zolix OmniFluo-990 (Zolix Instruments Co., Ltd., Beijing, China) with registration in the single photon counting mode at successive time intervals and automatic adjustment of the measured emission intensity was used for the registration of photoluminescence (PL) spectra. Measurements were carried out for 10^−5^ M solutions in the 10 mm quartz cuvette; the measurement geometry was 90°. All measurements were carried out at RT.

In regards to dynamic light scattering (DLS), hydrodynamic diameters of the NPs in water were determined by DLS at 20 °C using a Microtrac Zetatrac instrument (Microtrac Inc., York, PA, USA) equipped with a 4 mW He−Ne solid-state laser with 780 nm wavelength. Scattered light was detected at 180° in a controlled reference self-beating mode, and the particle size was calculated from the power spectrum analysis of the Doppler-shifted signal over several runs. For every tested sample, 5 runs each of 60 s duration were performed. The sphere-equivalent hydrodynamic diameter of the nanoparticles was calculated from the particle diffusion coefficient via the Stokes−Einstein equation, using a solution viscosity for liquid media at 20 °C.

In regards to cryo-TEM, a sample (3 µL) was placed onto a lacey carbon-supported copper grid (300 mesh), treated previously with air plasma to make it hydrophilic. An Excess of the sample was removed by blotting the grid for 1 s. Then the grid with the sample was plunged into liquid ethane (automated plunging system, Vitrobot FEI, Hillsboro, OR, USA). As prepared sample was transferred in liquid nitrogen to the TEM (Transmission electron microscope Tecnai G212 SPIRIT, FEI, Hillsboro, OR, USA).

In regards to flow cytometry, human breast carcinoma Sk-Br-3 cells were plated on 6-well plates (10^6^ cells per well) and incubated overnight. Then, 10 μg/mL of TTDCV and TTInd NPs were added to the cells in full DMEM media for 1 h, after which the cells were washed in phosphate buffer and detached with trypsin solution (0.25%). Fluorescence was measured on a channel corresponding to FITC fluorescence, and at least 20,000 events were examined for each sample.

In regards to confocal microscopy, human breast carcinoma Sk-Br-3 cells were plated on 8-well microscope slide flasks (10^5^ cells per well) and incubated overnight. Then, 10 μg/mL of TTDCV and TTInd NPs were added to the cells in complete DMEM medium for 30 min, after which the medium was replaced and the cells were irradiated at a wavelength of 530 nm, with 1 J/cm^2^. The cells were then washed in phosphate buffer to remove unbound substances and fixed in 4% paraformaldehyde. The nuclei were additionally stained with 10 µM Hoechst 33342. Fluorescence was analyzed at wavelengths of 405 nm (Hoechst 33342) and 488 nm and 543 nm (dyes) using a Leica TSP SPE confocal laser scanning system (Leica, Wetzlar, Germany).

In regards to the cytotoxicity study, human breast carcinoma cells Sk-Br-3 and MCF-7 were seeded in 96-well plates (10^5^ cells per well) in complete DMEM medium. The next day, the medium in some wells was replaced with 100 μL of fresh medium containing TTDCV or TTInd NPs in the concentration range of 100–0.2 μg/mL (total TRI points + control). Cells were incubated with NPs for 1.5 h, after which they were irradiated at 530 nm wavelength for 4 min (light dose ~1 J/cm^2^); non-irradiated cells were used as control. Cell viability was assessed after 72 h by MTT method. For this purpose, MTT solution (0.5 mg/mL) was added to each well. After 2 h, the medium was removed, and 100 μL of DMSO (99%) was added. Absorbance was recorded at 565 nm using an Infinite M Nano reader (Tecan, Männedorf, Switzerland). The IC_50_ values were determined using GraphPad Prism 10.3.0 software.

In regards to the intracellular ROS generation study by flow cytometry (CellROX™ Deep Red staining), human breast adenocarcinoma MCF-7 cells were plated on 12-well plates (2 × 10^5^ cells per well) and incubated overnight. Then, 10 μg/mL of TTDCV and TTInd NPs were added to the cells in full DMEM media for 1 h, after which the cells were washed in phosphate buffer and stained with 5 µM of CellROX DeepRed in DPBS for 30 min. Then the cells were irradiated with 530 nm, with total light dose of 1 J/cm^2^. After that, cells were detached with trypsin solution (0.25%). Non-irradiated cells were used as controls. Hydrogen peroxide (25 mM) was used as positive control. Ascorbic acid (0.5 mM) was used as an antioxidant. Fluorescence was measured on a channel corresponding to APC700 fluorescence, and at least 10,000 events were examined for each sample.

In regards to the NP stability assay, TTDCV and TTInd NPs (100 μg/mL) were stored in sealed glass vials at room temperature. At predetermined time intervals, the hydrodynamic size of the NPs was measured by DLS. Experiments were performed in triplicate.

### 3.4. Experimental Procedures

In regards to the preparation of NPs, 50 µL of a DMSO solution containing 1 mg of TTDCV, or TTInd, and 2 mg of poly(ethylene glycol)-block-polylactide methyl ether (PEG-b-PLA), or Pluronic F-127, was poured into 3 mL of deionized warm water with intense stirring, and colloidal solution was obtained and used directly. All dispersions were treated with ultrasound in an ultrasonic bath for 1 min to disassemble nanoparticle agglomerates. Hydrodynamic diameters of all obtained NPs were determined using the DLS method.

In regards to the NP stability assay, 100 µg/mL solutions of TTDCV NPs and TTInd NPs were placed in sealed disposable cuvettes for DLS measurements and incubated at room temperature. At predetermined time intervals, the hydrodynamic size of nanoparticles was measured by DLS. Experiments were performed in triplicate.

In regards to the computational details, all quantum chemical calculations were performed with the ORCA program package (v. 6.0.1) [71]. For both compounds, a conformational search was performed utilizing the ORCA’s GOAT (Global Optimizer Algorithm) [72,73] module at the GFN2-xTB [74] level of theory followed by ensemble optimization at the r2SCAN-3c [75] level. The geometries were verified as minima in the absence of imaginary frequencies. For further calculations, one conformer with the lowest free energy value was selected for each molecule (Table S1).

Geometries and Hessians of the S_0_, S_1_, T_1_ states for both molecules were obtained at the (TD)-PBE0-D4/def2-SVP [76] level of theory starting from the r^2^SCAN-3c S_0_-geometries. In both cases, a single low-frequency (>−30 cm^−1^) imaginary mode was found for S_1_ state, which could not be removed by increasing the optimization threshold and grid, and it was turned positive for further applications. Single-point and TDDFT-calculations were performed at the PBE0-D4/def2-TZVPPD level of theory. The frontier orbital energies were obtained as electron affinity/ionization potentials in accordance with the approach based on the Koopmans theorem [77] with the inclusion of CPCM (Acetonitrile) solvation model. The relative energies of the states obtained at this level are given relative to the zero-point energies (ZPE). Electrostatic potential maps were constructed with the Multiwfn program (v.3.8) [78,79,80] based on PBE0-D4/def2-TZVPPD//PBE0-D4/def2-SVP calculation and plotted with VMD (v. 1.9.3) [81]. Frontier orbitals were visualized with the Iboview program (v. 20211019) [82].

Rate constants were performed with ORCA’s Excited State Dynamics module [83] at the PBE0-D4/def2-TZVPPD level of theory. Each calculation considered the Duschinsky rotation and the Herzberg–Teller mechanism contribution. In the case of intersystem crossing (ISC) rate constant calculation, the Herzberg–Teller mechanism was excluded due to a strong overestimation (with an order of magnitude 10^10^). The resulting ISC rate constants are obtained as sums of ISC rates from the S_0_ level to T_1_ sublevels with different angular momentum (−1, 0, +1).

In regards to the photothermal effect and PCE calculation, the aqueous solutions of TTDCV and TTInd NPs with different concentrations were continuously exposed to a 530 nm laser with the power density of 0.5 W/cm^2^ for 6 min), and then NP dispersions were allowed to cool to room temperature. The temperature of the dispersions was monitored during the whole process.

In regards to thermal conversion efficiency evaluations, thermal measurements were conducted on the 0.1 mL aliquots of water solutions under study TTDCV and TTInd NPs; water was used as a reference. Substances in 1.5 mL Eppendorfs were subsequently placed in a holder above optical table level and illuminated at middle-column height with a collimated laser light (ytterbium fiber laser IPG IRE-Polus VLM-536-5 at 2nd harmonic with wavelength = 536 nm, Power = 290 mW, measured with Thorlabs powermeter PM100D, beam diameter 3 mm) until temperature stabilization with subsequent cooling to room temperature (21 ° C). Temperatures were measured with an IR camera Xenics Gobi-384-GigE-7098 (Xenics Infrared Solutions, Leuven, Belgium) placed perpendicular to both the laser beam and Eppendorf at the distance of ~25 cm so that the Eppendorf was imaged clearly at rate 10 fps. Numerical results were then gathered with the Xeneth v2.6.0.309 software.

Thermal coefficients were evaluated in the following way, adapted from [60] for the specific case of Eppendorf-based measurements.

Heat balance equation was considered:

$$m_{w}c_{w}\frac{dT}{dt}+\;m_{ep}c_{ep}\frac{dT}{dt}=W_{in,sample}+\;W_{in,\;ref}-W_{out}$$

where $m_{w},\;m_{ep}$ are water and Eppendorf masses, $c_{w},c_{ep}$ are their specific heat capacitances, $W_{in,sample},\;W_{in,\;ref}\;$ are heat power sources terms, dissipated by sample and reference (which is both water and Eppendorf), and $W_{out}$ is a heat loss term towards relatively cold room. Consideration of heat stored in the Eppendorf while warming and cooling samples was found important since at the mass of the aliquot being $m_{w}=$ 0.1 [mg] and $c_{w}=4.18$ [J/gC], the numbers for the Eppendorf are $m_{ep}$ = 0.11 [mg] (weighted after cutting Eppendorf at 0.1mL liquid column height) and $c_{ep}$ = 1.7 [J/gC] (polypropylene), which means that neglecting the term $m_{ep}c_{ep}\frac{dT}{dt}$ would cost a systematic underestimation of stored heat term $m_{w}c_{w}\frac{dT}{dt}$ by mepcepmwcw , resulting in subsequent underestimation of the thermal conversion coefficient by ~30% (for example, you may get 0.17 instead of 0.25). So we find it important to keep this term while neglecting spatial inhomogeneity of temperatures by averaging the temperature over most of the water–Eppendorf volumes during the IR data evaluation step.

Next, we can white down expression for $W_{in,sample}$ since according to the Beer–Lambert law the light power made through the Eppendorf is

$$I=I_{0}10^{-A_{\lambda }}$$

where $A_{\lambda }=l\kappa_{\lambda }n$ is absorbance, depending on length l the light makes through the Eppendorf (found to be 3.2 mm at the center of laser beam), sample concentration n and extinction coefficient $\kappa_{\lambda }$ is of the sample at laser wavelength, and $I_{0}$[W] is incident laser power. Then the absorbed power is

$$I_{0}-I=I_{0}(1-10^{-A_{\lambda }})$$

Part of that power converted to the heat is determined by thermal conversion coefficient η:

$$W_{in,sample}=\eta *I_{0}(1-10^{-A_{\lambda }})$$

Term $W_{in,\;ref}$ was found to be neglectable in our setting at 535 nm in comparison with $W_{out}\;$ since at a given heat loss rate $W_{out}\;$ the reference sample (water in Eppendorf) was not heating at all (see Figure 5), which is due to relatively cold room conditions, although in general the reference case can be treated in the same way as the general one.

The heat loss term $W_{out}$ was treated in linear approximation:

$$W_{out}=hS(T-T_{surr})$$

where *h* is heat transfer coefficient, *S* is surface through which heat transfer appears, T= T(t) or the current temperature of sample depending on time, and $T_{surr}$ is the surrounding temperature. Both temperatures were found by the IR measurements in regions of interest (*T* inside the Eppendorf, $T_{surr}$ on the close background).

If we plug in everything in the heat balance equation, we get

$${(m}_{w}c_{w}+m_{ep}c_{ep})\frac{dT}{dt}=\eta *I_{0}(1-10^{-A_{\lambda }})-hS(T-T_{surr})$$

which can be easily solved in two-step process, if a steady state *T* is reached during illumination, which holds in our case.

First, the steady state solution excludes any changes in temperature so that $\frac{dT}{dt}=0$ and $T\;=T_{max}$, and so from the heat balance equation we get

$$\eta =\frac{hS\left(T_{max}-T_{surr}\right)}{I_{0}\left(1-10^{-A_{\lambda }}\right)}$$

where we now know everything except hS, which can be found from the second special case, when laser source term is off $W_{in,sample}=0$ and temperature gradually decreases from $T_{max}$ to $T_{surr}$:

$${(m}_{w}c_{w}+\;m_{ep}c_{ep})\frac{dT}{dt}=\;-hS(T-T_{surr})$$

This equation can be solved to get

$$T(t)=T_{surr}+\left(T_{max}-T_{surr}\right)10^{-\frac{hS\left(t-t_{0}\right)}{m_{w}c_{w}+m_{ep}c_{ep}}}$$

where $t_{0}$ [s] is laser switching-off time. From this, hS can be found by fitting the cooling data using the expression below:

$$\frac{{T\left(t\right)-T}_{surr}}{T_{max}-T_{surr}}\;=10^{-\;\frac{hS\left(t-t_{0}\right)}{m_{w}c_{w}+\;m_{ep}c_{ep}}}$$

In logarithmic coordinates, it is the following:

$$log\frac{{T\left(t\right)-T}_{surr}}{T_{max}-T_{surr}}\;=-\;\frac{hS\left(t-t_{0}\right)}{m_{w}c_{w}+\;m_{ep}c_{ep}}=\;\frac{hSt_{0}}{m_{w}c_{w}+\;m_{ep}c_{ep}}-\frac{hS}{m_{w}c_{w}+\;m_{ep}c_{ep}}*\;t$$

So we get *hS* from linear approximation of $log\frac{{T\left(t\right)-T}_{surr}}{T_{max}-T_{surr}}$ as a function of t. Knowing *hS*, we can find η.

## 4. Conclusions

In summary, D–A molecules were successfully engineered into spherical NPs via two approaches: nanoprecipitation from DMSO solution into water, yielding the polymer-free NPs dispersion, and encapsulation within two amphiphilic polymer matrices PEG-*b*-PLA or Pluronic^®^ F-127. The resulting NPs exhibited a core–shell structure and a tunable mean diameter ranging from 66 to 139 nm, contingent upon the polymer carrier. The molecular structure of the D–A molecules was shown to influence the optical properties and biological activity of the resulting NPs. At the same time, the choice of polymer matrix strongly affected NP size (66–139 nm), colloidal stability, and cellular uptake, which in turn determined their cytotoxicity. The NPs exhibited remarkable photothermal and photodynamic activity against Sk-Br-3 and MCF-7 human breast cancer cell lines, as demonstrated by intracellular ROS generation and efficient heating under low-dose irradiation. In general NPs with Pluronic^®^ F-127 yielded smaller, more stable NPs with enhanced cytotoxicity (PI up to 26) under light irradiation. The results emphasize that the therapeutic potential of D–A-based nanomaterials depends not only on the molecular scaffold but also on the optimization of NPs formulation, including polymer type and physicochemical parameters. These insights provide a valuable framework for developing advanced nanoplatforms for combined photodynamic and photothermal cancer therapy.

Despite the promising in vitro performance of the developed NPs, several limitations should be acknowledged. The present study focused on proof-of-concept validation of the relationship between molecular structure, encapsulation strategy, and phototherapeutic activity. Additional investigations are required to evaluate biodistribution, pharmacokinetics, long-term biocompatibility, and therapeutic efficacy in vivo. Furthermore, although efficient phototherapy was achieved under green-light irradiation, future molecular engineering efforts aimed at shifting absorption toward the NIR region may further enhance clinical applicability.

## Figures and Tables

**Table 1:** Optical properties of TTDCV and TTInd in THF solution, polycrystalline film, and different NP forms.

Compound	Solution		Film		Compound	NPs		
	*λ*_abs_, nm	*λ*_onset_, nm	*λ*_abs_, nm	*λ*_onset_, nm		*λ*_abs_, nm	*λ*_onset_, nm	d_av_, nm
TTDCV	487	550	520	618	TTDCV	505	617	106
					TTDCV-PP	504	610	96
					TTDCV-Pl	494	593	66
TTInd	511	583	532	635	TTInd	523	653	93
					TTInd-PP	530	661	139
					TTInd-Pl	511	627	69

λ_abs_ is an absorption maximum; λ_onset_ is an onset of an absorption spectrum (high energy region); and d_av_ is the average hydrodynamic diameter of NPs.

**Table 2:** The IC_50_ and PI values for cell viability of human breast carcinoma Sk-Br-3 and MCF-7 cells after incubation with TTDCV and TTInd NPs (MTT assay with a 72 h incubation).

Compound	Sk-Br-3 Cells			MCF-7 Cells		
	IC_50_, µg/mL		PI	IC_50_, µg/mL		PI
	Non-Irradiated	Irradiated		Non-Irradiated	Irradiated	
TTDCV	33.00 ± 2.55	6.10 ± 0.36	5.4	9.30 ± 1.13	3.81 ± 0.27	2.4
TTDCV-Pl	17.87 ± 1.26	1.66 ± 0.15	10.8	9.57 ± 1.05	0.99 ± 0.08	9.6
TTDCV-PP	18.32 ± 1.67	1.42 ± 0.22	12.9	8.89 ± 1.18	2.25 ± 0.16	4.0
TTInd	~100	~100	~1.0	>100	>100	>1.0
TTInd-Pl	>100	4.56 ± 0.78	>21.9	100	6.45 ± 1.19	15.5
TTInd-PP	~100	54.09 ± 10.62	~1.85	>100	46.20 ± 5.29	>2.2

IC_50_—half-max inhibitory concentration; PI, phototoxicity index, calculated as the ratio of IC_50_ value under irradiation to IC_50_ value in the dark.

## Data Availability

The original contributions presented in this study are included in the article/Supplementary Materials. Further inquiries can be directed to the corresponding authors.

## Supplementary Materials

The following supporting information can be downloaded at https://www.mdpi.com/article/10.3390/ijms27135863/s1.

## Abbreviations

The following abbreviations are used in this manuscript:

D–A	Donor–acceptor
NPs	Nanoparticles
PEG-b-PLA	Poly(ethylene glycol)-block polylactide methyl ether
PEO-PPO-PEO, Pluronic^®^ F-127	Polyethylene oxide-polypropylene oxide
ROS	Reactive oxygen species
PSs	Photosensitizers
ICT	Intramolecular charge transfer
HOMO	Highest occupied molecular orbital
LUMO	Lowest unoccupied molecular orbital
NIR	Near-infrared
PDT	Photodynamic therapy
PTT	Photothermal therapy
EPR	Enhanced permeability and retention
DMSO	Dimethyl sulfoxide
DLS	Dynamic light scattering
FBS	Saline/fetal bovine serum
Cryo-TEM	Cryogenic transmission electron microscopy
DFT	Density functional theory
ISC	Intersystem crossing
PTCE	Photothermal conversion efficiency
PI	Phototoxicity indexes
