# Supplementary material for: The Role of Polymer Encapsulation in Optimizing Donor–Acceptor Organic Nanoparticles for Efficient Cancer Phototherapy
Source: Int J Mol Sci. 2026 Jun 29;27(13):5863. doi: 10.3390/ijms27135863 (PMC13361513; doi:10.3390/ijms27135863)
Supplement: Supplementary file 1 [file ijms-27-05863-s001.zip › ijms-4279128-supplementary.pdf]

SUPPLEMENTARY MATERIALS

for

The Role of Polymer Encapsulation in Optimizing Donor–Acceptor Organic Nanoparticles for Efficient Cancer Phototherapy

Yulia A. Isaeva[1], Dmitry O. Balakirev[1], Anastasia A. Vetyugova[1], Maxim E. Stepanov[2,3], Michael D. Khitrov[1], Nikita S. Saratovsky[1], Mikhail V. Zolotov[1], Tatyana V. Egorova[1,4], Polina A. Demina[3,4], Roman A. Akasov[1,3*], Yuriy N. Luponosov[1*]

[a] Enikolopov Institute of Synthetic Polymeric Materials of the Russian Academy of Sciences, Profsoyuznaya St. 70, Moscow 117393, Russia;

[b] Moscow Pedagogical State University, Malaya Pirogovskaya St. 29/7, building 1, Moscow 119991, Russia;

[c] Shemyakin & Ovchinnikov Institute of Bioorganic Chemistry of the Russian Academy of Sciences, Moscow 117997, Russia

[d] Petrovsky National Research Center of Surgery, Moscow 119991, Russia

*Corresponding author e-mail: luponosov@ispm.ru

roman.akasov@gmail.com

Contents

ELECTRONIC SUPPORTING INFORMATION ....SОшибка! Закладка не определена.

1. Experimental part **S2**

2. NMR Spectra **S3**

3. Properties of nanoparticles **S4**

4. In vitro ROS generation and cytotoxicity of NPs **S7**

1. Experimental part

Table S1. Conformers of the studied compounds and their relative free energies

TTDCV	
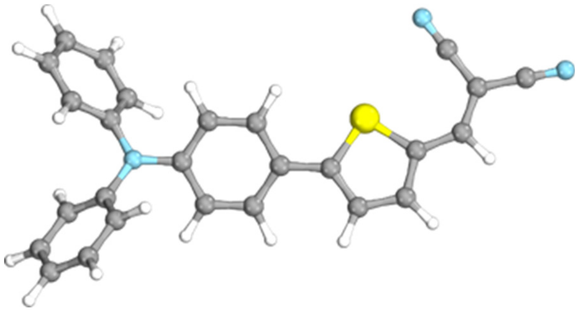	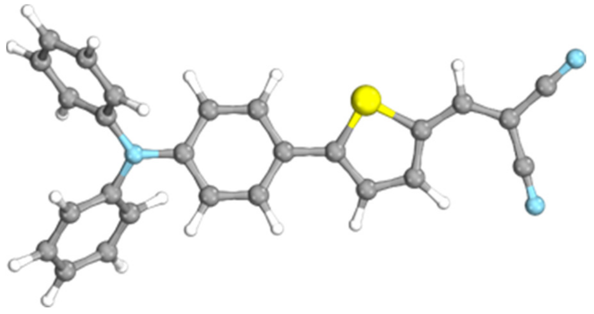
$\Delta G_{rel.}^{298K} = 0$	$\Delta G_{rel.}^{298K} = 0.86\ kcal \cdot mol^{-1}$
TTInd	
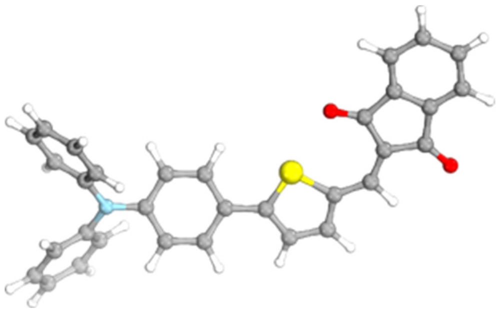	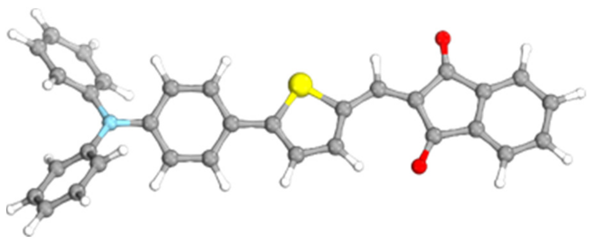
$\Delta G_{rel.}^{298K} = 0$	$\Delta G_{rel.}^{298K} = 0.69\ kcal \cdot mol^{-1}$

2. NMR Spectra

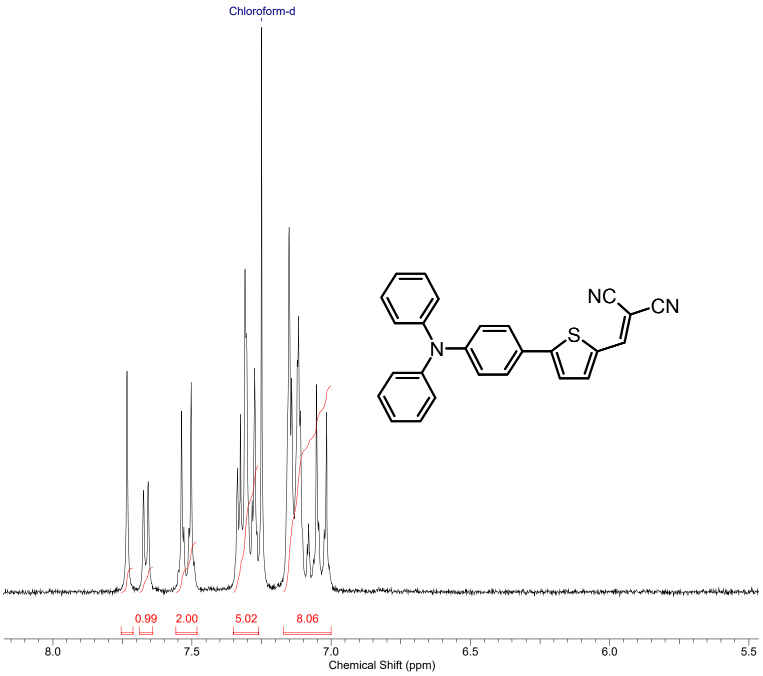

No.	Annotation	(ppm)
1	Chloroform-d	7.25

No.	(ppm)	Value	Absolute Value
1	[7.00 .. 7.17]	8.065	8.91430e+7
2	[7.26 .. 7.35]	5.020	5.54877e+7
3	[7.48 .. 7.56]	2.000	2.21071e+7
4	[7.64 .. 7.69]	0.986	1.08987e+7
5	[7.71 .. 7.75]	0.960	1.06144e+7

Figure S1. ^{1}H NMR spectrum of TTDCV.

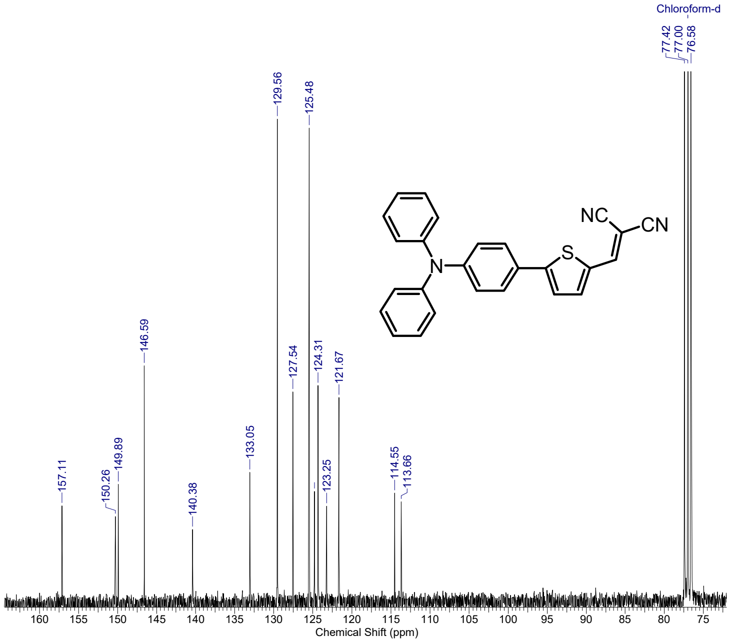

No.	(ppm)	(Hz)	Height
1	76.58	5780.0	0.9853
2	77.00	5811.8	1.0000
3	77.42	5843.9	0.9822
4	113.66	8579.1	0.1396
5	114.55	8646.0	0.1504
6	121.67	9183.1	0.2669
7	123.25	9302.3	0.1345
8	124.31	9382.7	0.2815
9	124.80	9419.8	0.1522
10	125.48	9470.6	0.5959
11	127.54	9626.5	0.2738
12	129.56	9778.9	0.6071
13	133.05	10042.6	0.1755
14	140.38	10595.7	0.1057
15	146.59	11064.0	0.3055
16	149.89	11313.6	0.1608
17	150.26	11341.3	0.1217
18	157.11	11858.5	0.1349

No.	Annotation	(ppm)
1	Chloroform-d	77.00

Figure S2. ^{13}C NMR spectrum of TTDCV.

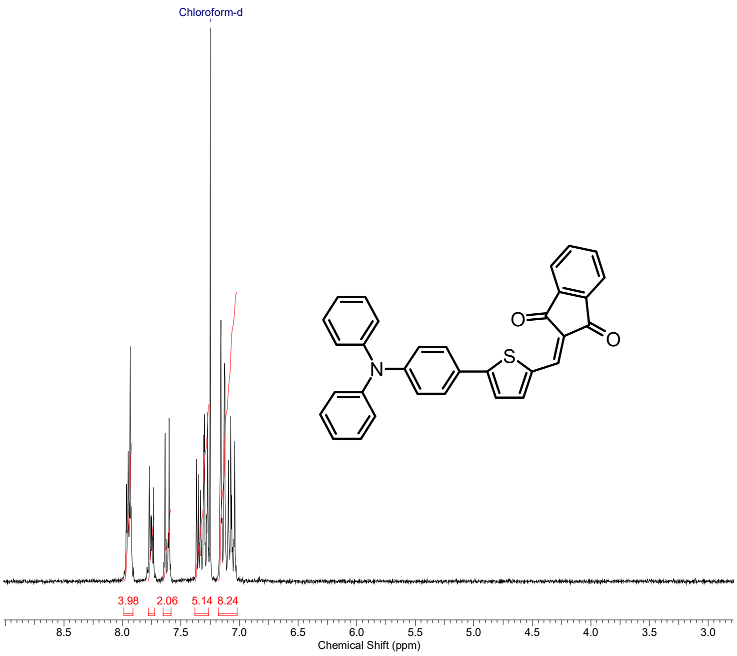

No.	Annotation	(ppm)
1	Chloroform-d	7.25

No.	(ppm)	Value	Absolute Value
1	[7.02 .. 7.18]	8.237	2.16868e+7
2	[7.26 .. 7.38]	5.140	1.35321e+7
3	[7.58 .. 7.65]	2.061	5.42702e+6
4	[7.73 .. 7.78]	1.847	4.86425e+6
5	[7.91 .. 7.99]	3.977	1.04709e+7

Figure S3. ^{1}H NMR spectrum of TTInd.

3. Properties of nanoparticles

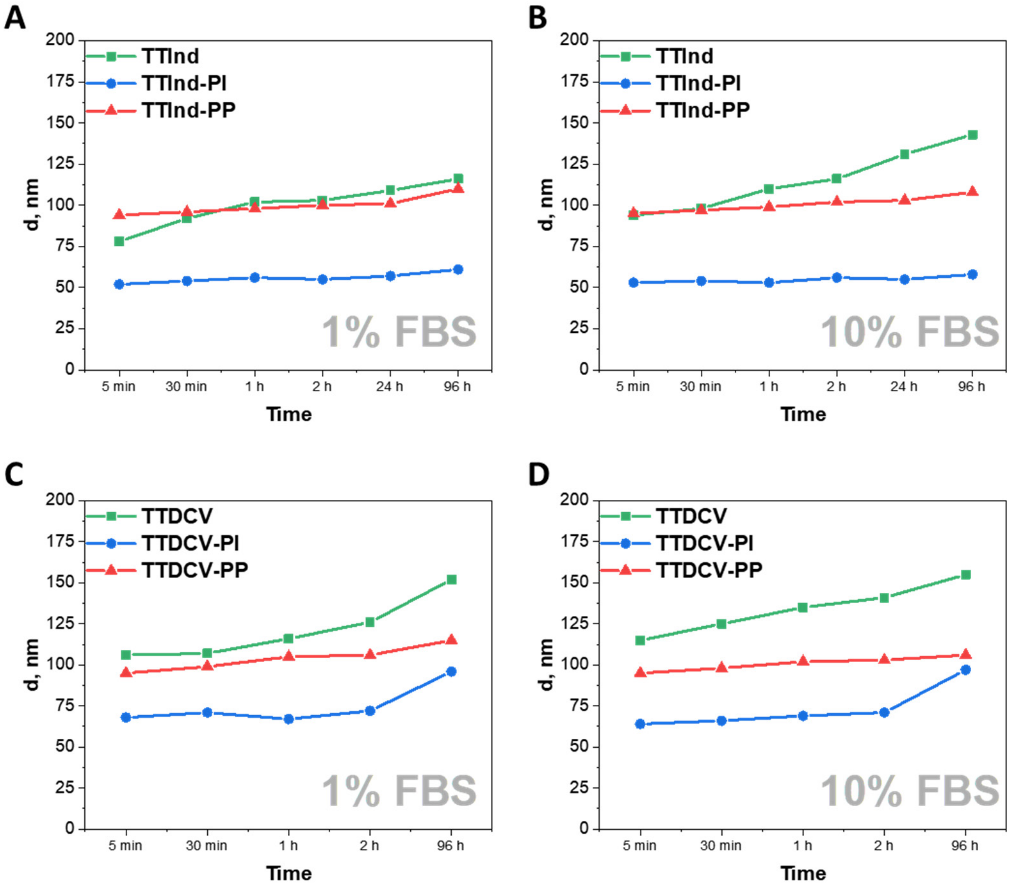

Figure S4. TTDCV and TTInd NPs size growth in saline/fetal bovine serum (FBS) mixtures containing 1% (A, C) and 10% (C, D) FBS obtained by DLS.

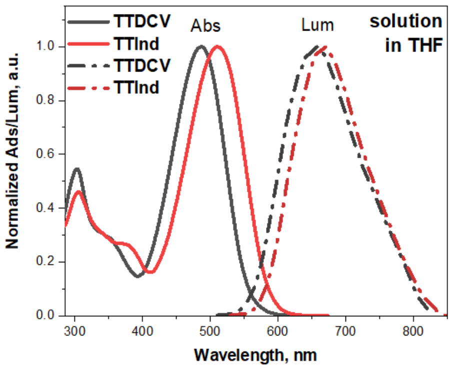

Figure S5. UV-vis absorption and photoluminescence spectra of TTDCV and TTInd in the diluted THF solutions.

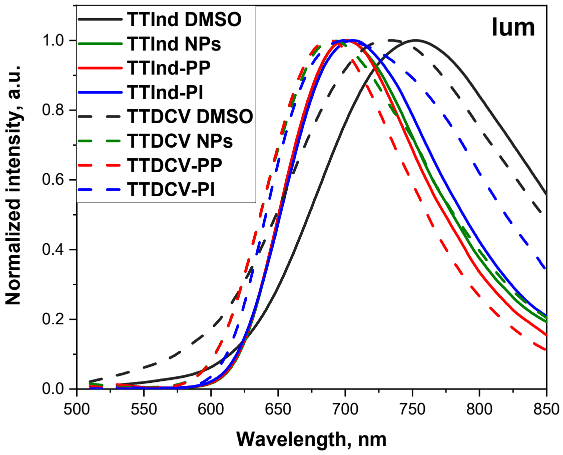

Figure S6. Normalized photoluminescence spectra of TTDCV and TTInd in the diluted DMSO solutions and NPs dispersed in water.

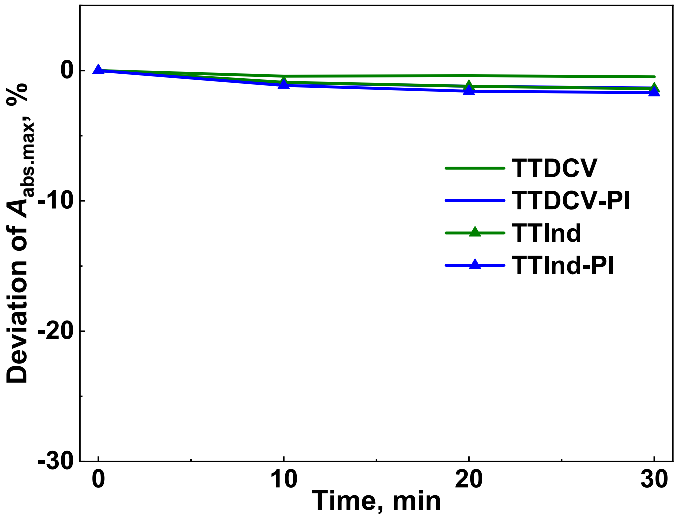

Figure S7. Change in intensity of absorption maximum during time for NPs (TTDCV, TTDCV-Pl, TTInd and TTInd-Pl, concertation: 13 mg/mL) under continuous 530 nm LED irradiation (150 mW/cm^2).

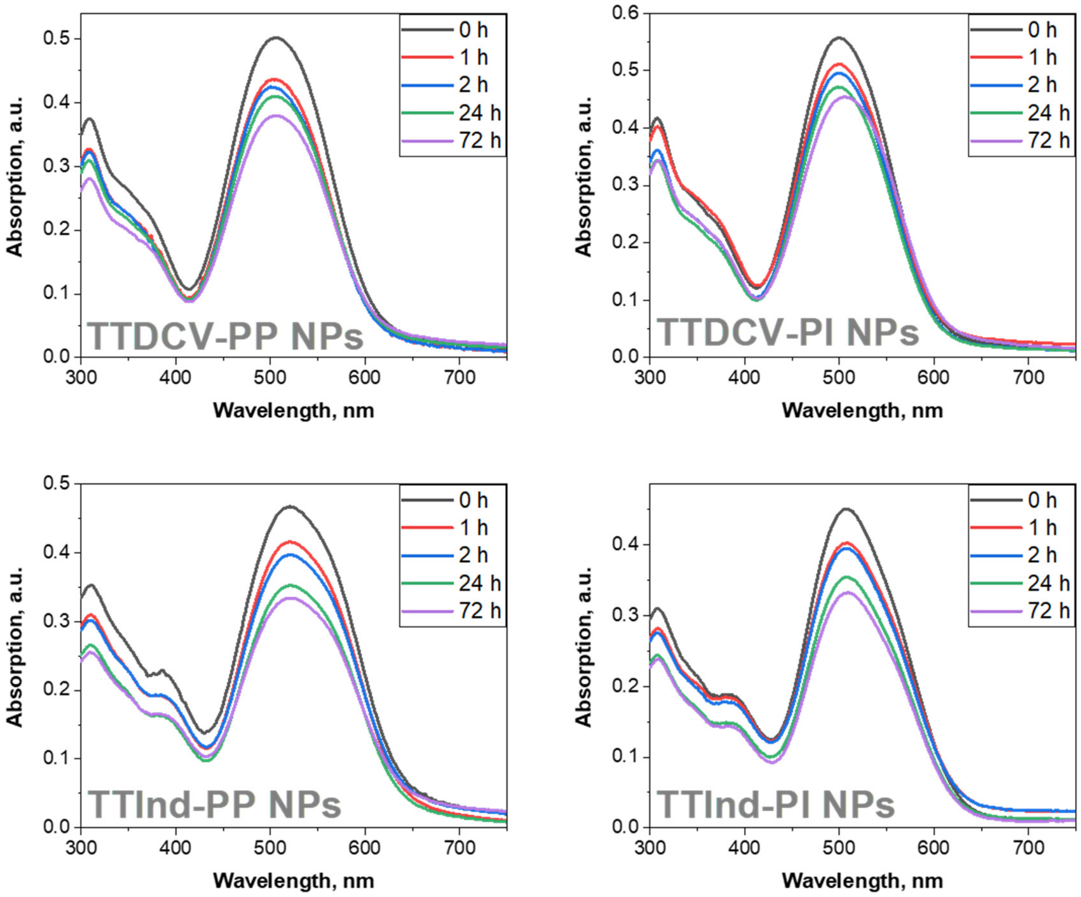

Figure S8. Time-dependent release profiles of TTDCV and TTInd from polymeric nanoparticles based on PEG–PLA (PP) and Pluronic (Pl). UV–Vis absorption spectra were recorded at 0, 1, 2, 24, and 72 h under physiological conditions. Progressive changes in absorbance intensity indicate dye release from the NPs matrices.

Table S2. Photothermal coefficient values calculated for TTDCV and TTInd NPs (20 µg/ml)

Compound	OD at 536 nm	T_{max}, Celsius	T_{surr}, Celsius	Thermal coefficient, %
TTInd-PP	1,2292	45,631	20,747	0,257
TTInd -Pl	1,207	48,011	20,687	0,286
TTInd	0,954	43,057	21,0785	0,267
TTDCV-PP	0,8144	41,704	21,228	0,278
TTDCV-Pl	0,8804	41,811	21,4495	0,260
TTDCV	1,0625	43,524	22,1025	0,239

4. In vitro ROS generation and cytotoxicity of NPs

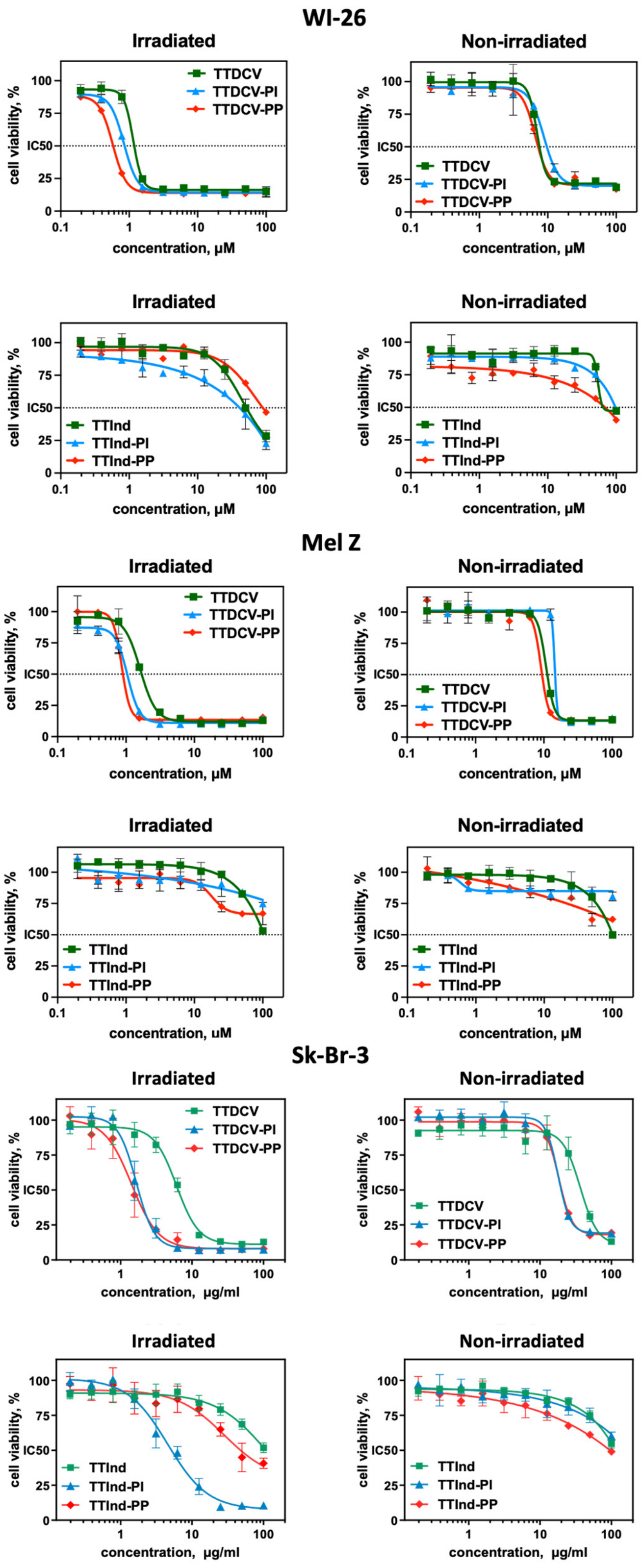

Figure S9. Cell viability of lung fibroblast WI-26, human melanoma Mel Z and human breast cancer Sk-Br-3 cells depending on the concentration of NPs (μg/ml) when irradiated with light (530 nm, 1 J/cm2) and in the dark; 72 h incubation; MTT assay, data are present as mean±SD.

Table S3. The IC_{50} and PI values for cell viability of lung fibroblast WI-26 and human melanoma Mel Z cells after incubation with TTDCV and TTInd NPs, MTT assay, 72 h incubation.

Compound	WI-26 cells			Mel Z cells		
	IC_{50}, µg/ml		PI	IC_{50}, µg/ml		PI
	Non-irradiated	*Irradiated*		*Non-irradiated*	*Irradiated*	
TTDCV	6.82 ± 0.77	1.14 ± 0.11	5.98	10.73 ± 1.93	1.61 ± 0.13	6.7
TTDCV-Pl	8.76 ± 1.19	0.84 ± 0.03	10.43	14.39 ± 2.05	1.05 ± 0.08	13.7
TTDCV-PP	6.58 ± 0.53	0.58 ± 0.02	11.34	9.03 ± 1.18	0.86 ± 0.07	10.5
TTInd	~100	49.86 ± 3.11	~2.0	~100	~100	~1
TTInd-Pl	>100	41.36 ± 2.48	>2.4	> 100	> 100	-
TTInd-PP	~100	~100	~1.0	>100	> 100	-

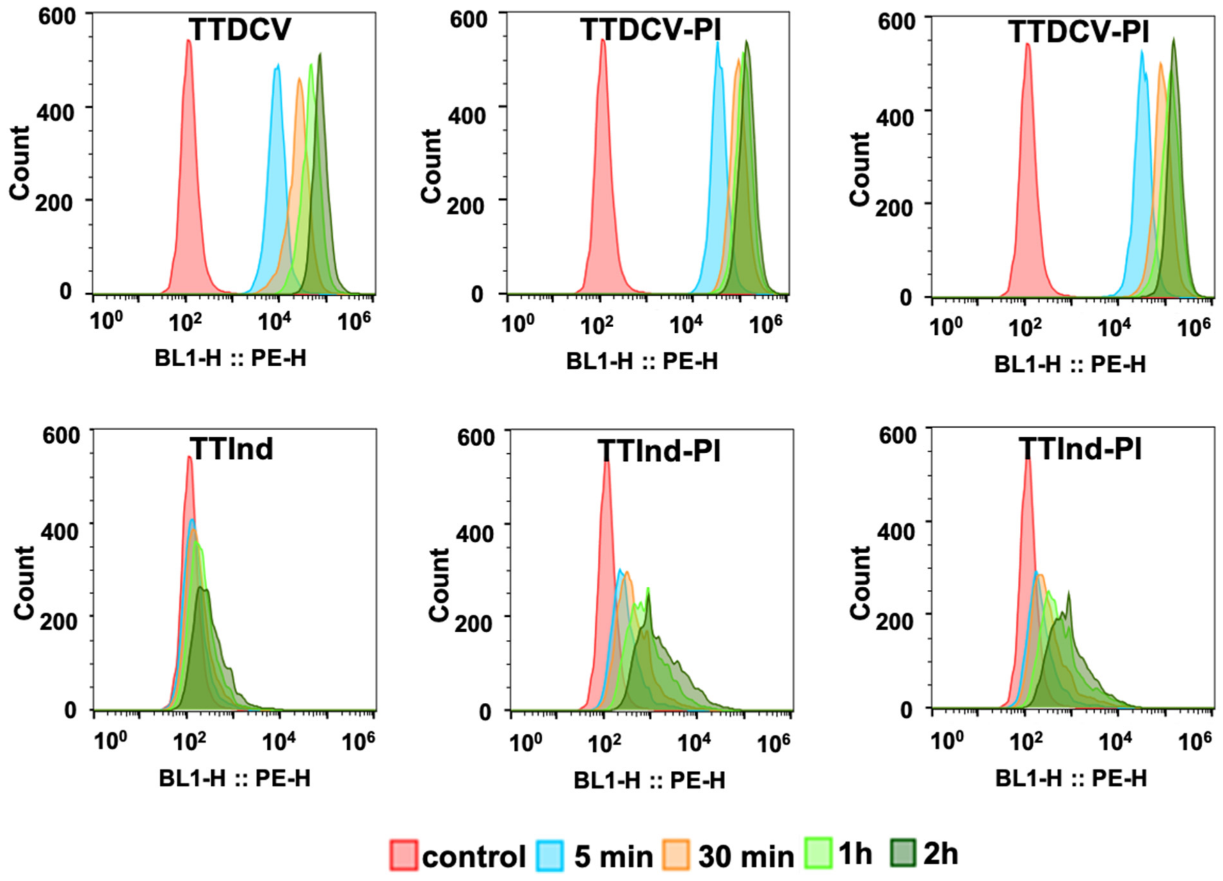

Figure S10. Intracellular accumulation of TTDCV and TTInd NPs in human breast carcinoma MCF-7 cells, 10 µg/ml, 5 min, 30 min, 1 h and 2 h incubation. Primary flow cytometry curves. Flow cytometry data, 20,000 events in each sample; data are the mean ± SD.

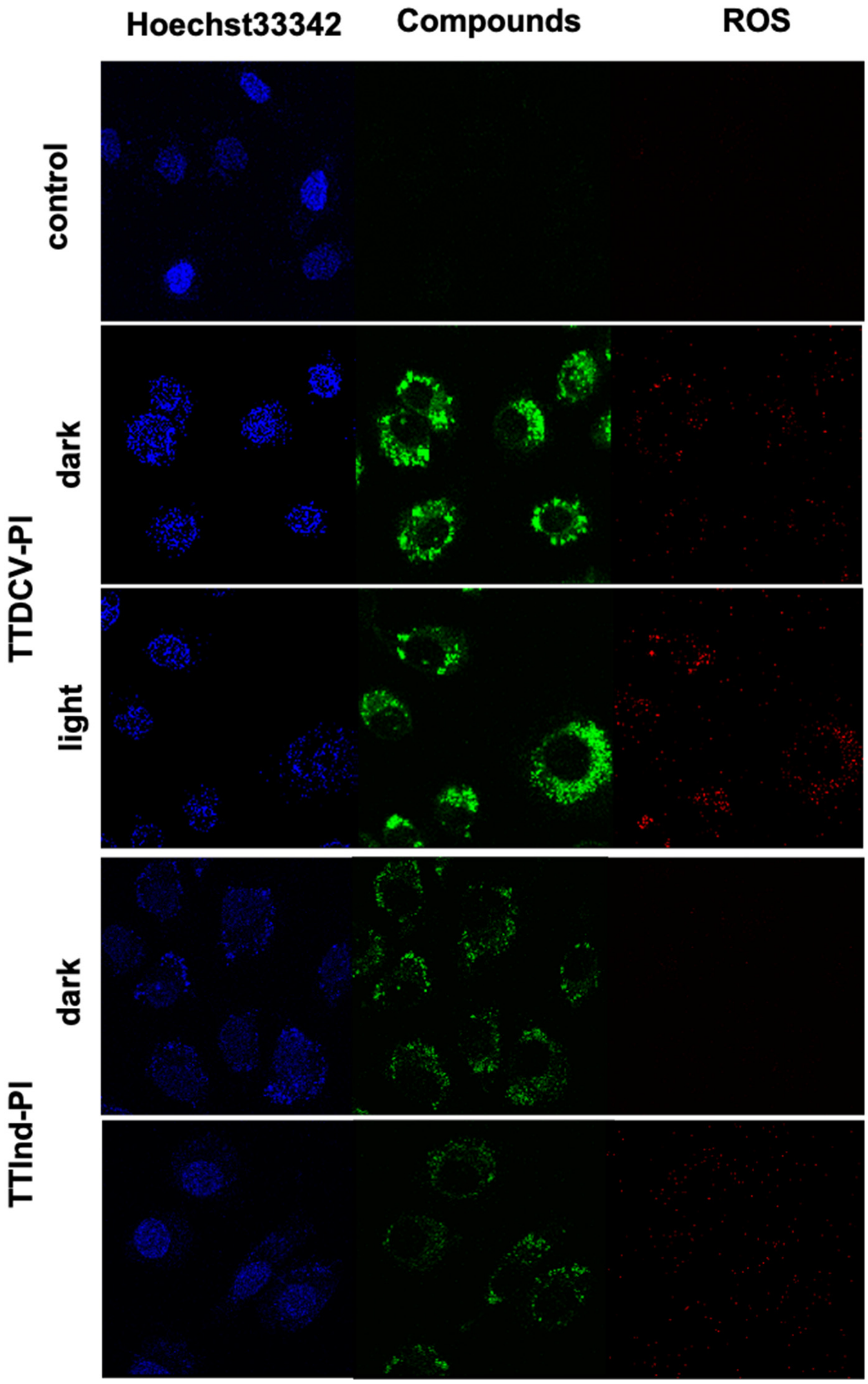

Figure S11. CLSM images of Sk-Br-3 tumor cells treated with NPs for 1.5 hours with or without subsequent irradiation with 530 nm LED. Confocal microscopy data, scale-bar 20 μm.

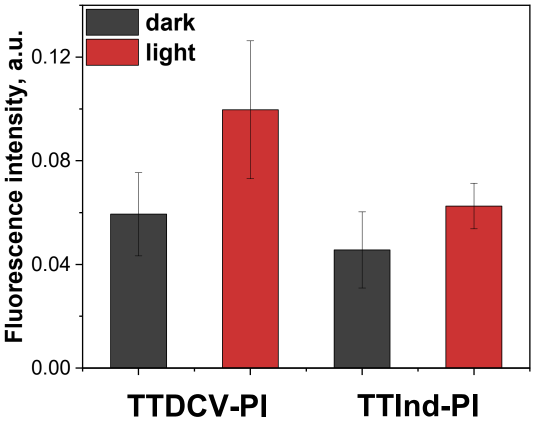

Figure S12. ROS-generatiom of **TTInd** and **TTDCV** NPs in human breast carcinoma Sk-Br-3 cells, 10 µg/ml; CLSM data.

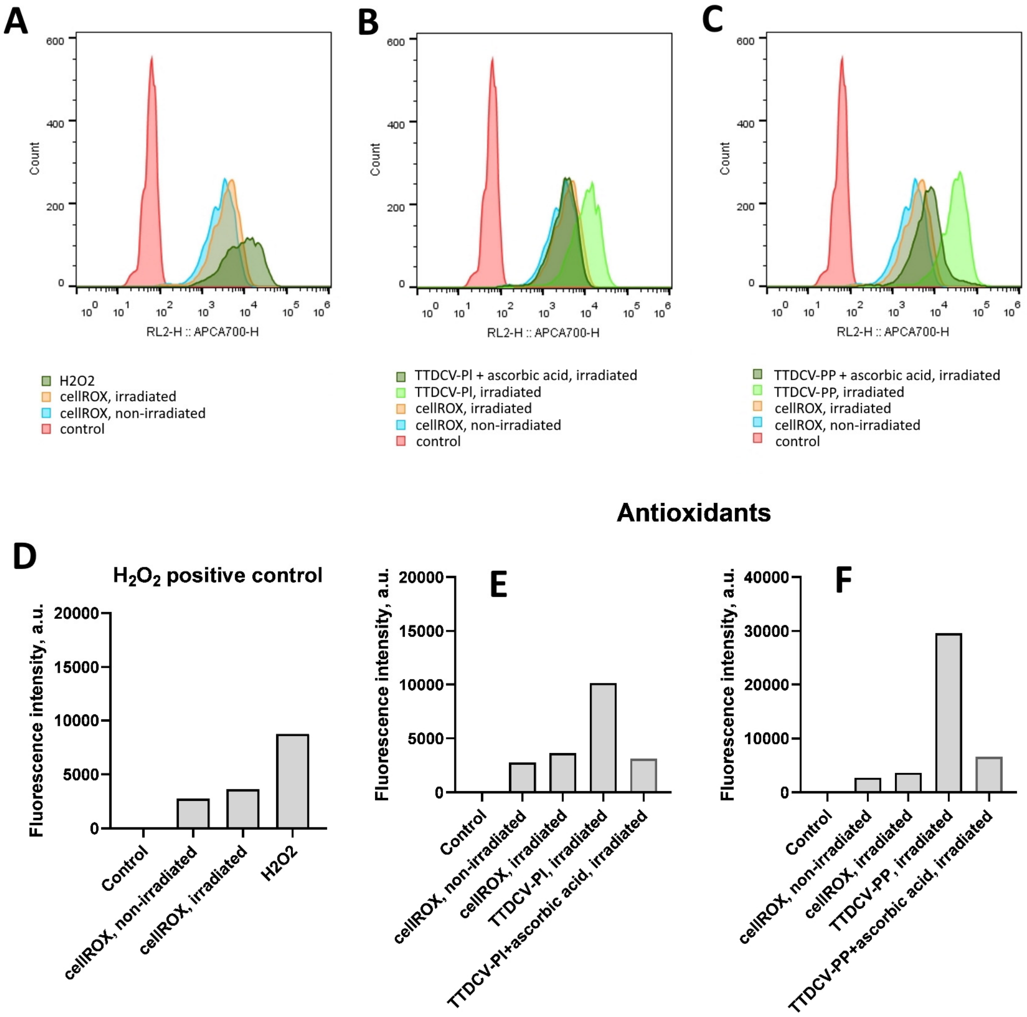

Figure S13. CellROX Deep Red staining of human breast adenocarcinoma MCF-7 cells: 25 mM H2O2 (A), TTDCV-PI in the presence of 0.5 mM ascorbic acid (B), TTDCV-PP in the presence of 0.5 mM ascorbic acid (C). Median values of CellROX Deep Red fluorescence (D-F). Flow cytometry data, 10,000 counts per each sample.

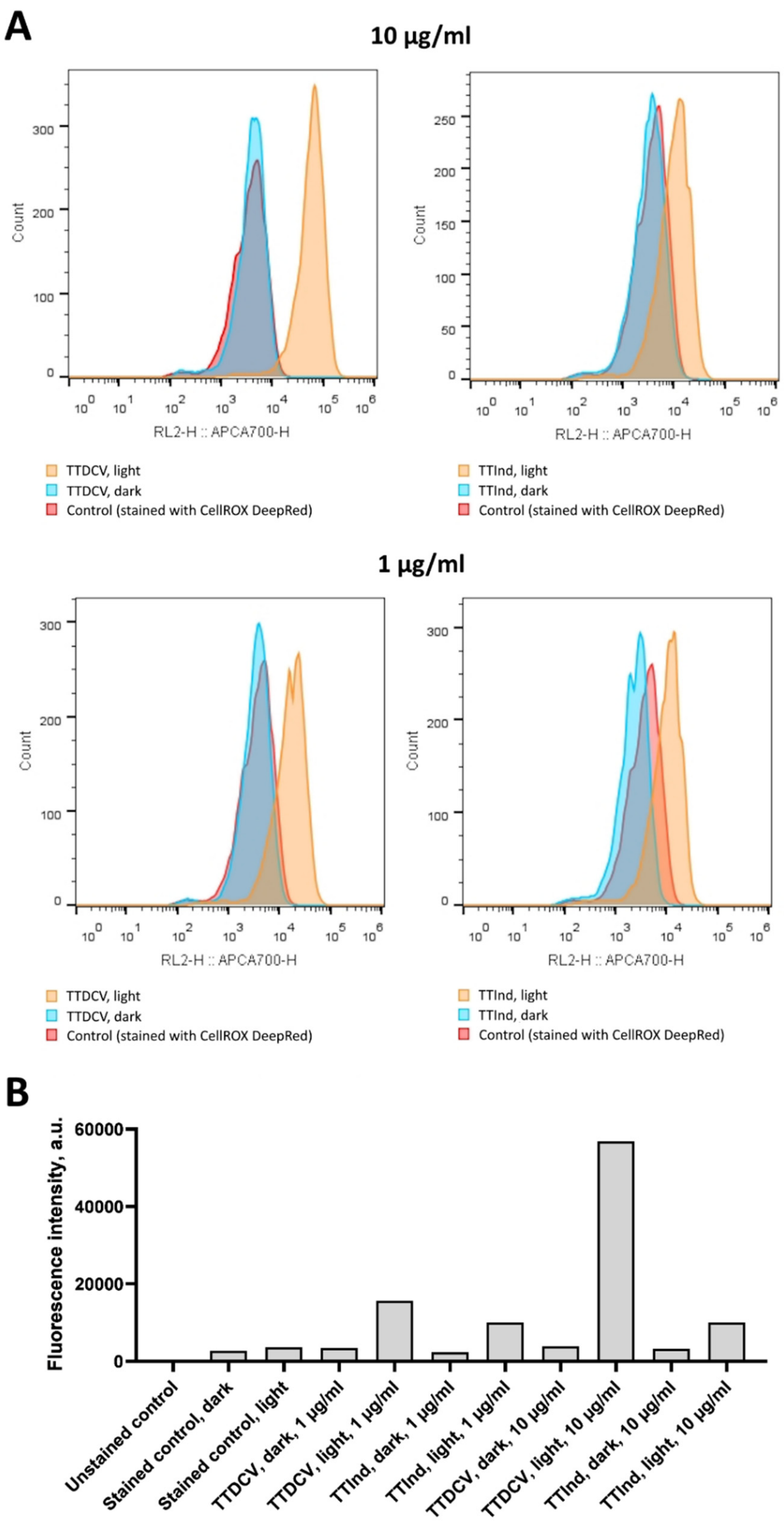

Figure S14. Intracellular ROS generation in human breast adenocarcinoma cells treated with TTDCV and TTInd, flow cytometry data. The fluorescent intensity curves (A) and the median values of the samples (B).
